# Roles of Metal Ions in MXene Synthesis, Processing and Applications: A Perspective

**DOI:** 10.1002/advs.202200296

**Published:** 2022-02-26

**Authors:** Yu Long, Ying Tao, Tongxin Shang, Haotian Yang, Zejun Sun, Wei Chen, Quan‐Hong Yang

**Affiliations:** ^1^ Joint School of National University of Singapore and Tianjin University International Campus of Tianjin University Binhai New City Fuzhou 350207 China; ^2^ Department of Chemistry National University of Singapore 3 Science Drive 3 Singapore 117543 Singapore; ^3^ Nanoyang Group State Key Laboratory of Chemical Engineering School of Chemical Engineering and Technology Tianjin University Tianjin 300072 China; ^4^ Key Laboratory of Resource Chemistry of Ministry of Education Shanghai Key Laboratory of Rare Earth Functional Materials Department of Chemistry Shanghai Normal University Shanghai 200234 China; ^5^ Department of Physics National University of Singapore 2 Science Drive 3 Singapore 117542 Singapore

**Keywords:** application, metal ion, MXenes, processing, synthesis

## Abstract

With a decade of effort, significant progress has been achieved in the synthesis, processing, and applications of MXenes. Metal ions play many crucial roles, such as in MXene delamination, structure regulation, surface modification, MXene composite construction, and even some unique applications. The different roles of metal ions are attributed to their many interactions with MXenes and the unique nature of MXenes, including their layered structure, surface chemistry, and the existence of multi‐valent transition metals. Interactions with metal ions are crucial for the energy storage of MXene electrodes, especially in metal ion batteries and supercapacitors with neutral electrolytes. This review aims to provide a good understanding of the interactions between metal ions and MXenes, including the classification and fundamental chemistry of their interactions, in order to achieve their more effective utilization and rational design. It also provides new perspectives on MXene evolution and exfoliation, which may suggest optimized synthesis strategies. In this respect, the different effects of metal ions on MXene synthesis and processing are clarified, and the corresponding mechanisms are elaborated. Research progress on the roles metal ions have in MXene applications is also introduced.

## Graphical abstract

Interactions with metal ions play crucial roles in the development of MXenes. This review discusses in details the fundamental chemistry of these interactions, including intercalation, crosslinking and etching, and presents how these interactions affect MXene synthesis, processing and applications, which yields the approaches for the rational designs, optimized synthesis and potential applications of MXene‐based materials.

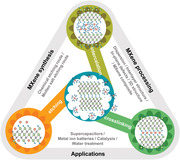

## 1 Introduction

Since graphite was delaminated to form graphene, there has been tremendous research interest on 2D materials in academia and industry, due to its remarkable physical and electronic properties. In recent decades, various other 2D materials besides graphene have been explored including transition metal dichalcogenides (TMDs), metal carbides/nitrides, layered double hydroxides (LDHs), boron nitride (h‐BN), graphene‐like single elements (e.g., black phosphorus), etc.^[^ ^1^, ^2^, ^3^, ^4^ ^]^ They typically have strong in‐plane bonding and weak out‐of‐plane interactions, with very large exposed surface areas. Their properties can be effectively changed by altering their composition, interlayer structure, and surface chemistry, which gives them potential use in many fields, such as optoelectronics, catalysis, energy, sensors, and biomedicine.^[^ ^5^, ^6^ ^]^

MXenes are a range of new 2D transition metal carbide/nitride materials with a functional structure, which were first discovered in 2011^[^ ^7^ ^]^ and well defined in 2012.^[^ ^8^ ^]^ This category of 2D materials shares the general formula M*_n_*_+1_X*_n_* T*_x_* (*n* = 1–3), where M is a transition metal (e.g., Ti, V, Mo, Cr), and X is carbon and/or nitrogen, with surface terminations indicated by T (e.g., ‐O, ‐OH, ‐F, ‐Cl, ‐Br, ‐S, ‐NH, ‐Se, ‐Te). The presence of single or mixed terminations in MXenes is determined by their synthesis or processing.^[^ ^9^, ^10^, ^11^ ^]^ In general, they are synthesized by selectively etching A layers from MAX phases, where A is a group of 13 or 14 elements (e.g., Al, Si, Ga), and M and X are the same as for the corresponding MXene. The first and the most studied MXene is Ti_3_C_2_T*_x_* (T = ‐O, ‐OH, ‐F), which was first produced in 2011 with a hydrofluoric acid (HF) solution as the etchant.^[^ ^7^ ^]^ The molten Lewis acid salt route enables other surface groups (i.e., ‐Cl, ‐Br, ‐I) to terminate the MXene surface.^[^ ^12^, ^13^ ^]^ Further termination substitution by ‐NH, ‐S, ‐Se, ‐Te, or complete removal can be achieved by substitution reactions. Modification of the surface chemistry enables fine‐tuning of the band gap as well as other properties, such as electrical, optical, and optoelectronic.^[^ ^14^, ^15^, ^16^, ^17^, ^18^ ^]^ For example, Liu et al. indicated that, compared with the bare surface, ‐O terminations tend to increase the work function of MXenes, while ‐OH terminations tend to cause a decrease. The tendency produced by ‐F terminations depends on the specific structure of the MXenes.^[^ ^15^ ^]^ A superconducting transition behavior has been reported at low‐temperatures for ‐S (or ‐Se, ‐NH) terminated Nb_2_C, while no such a behavior occurs for Nb_2_CO*_x_*.^[^ ^14^ ^]^ Till now, more than 30 MXenes with different compositions have been discovered experimentally, and over 100 different MXenes have been theoretically predicted. The M sites can also be filled by two or more transition metals to form solid solutions or ordered structures. This unique structure gives MXenes attractive electronic, optical, and magnetic properties. A transition metal carbide core gives MXenes metallic conductivity (20 000 S cm^−1^ for Ti_3_C_2_T*_x_*), and multi‐valent transition metals ensure good redox activity. The abundant surface terminations provide active sites, solution dispersion ability, and the ability to change properties. Through strategic design and controlled synthesis, MXenes have proven to be promising in various applications, such as supercapacitors, rechargeable metal‐ion batteries, catalysis, electromagnetic interference (EMI) shielding, and water treatment.^[^ ^19^, ^20^, ^21^ ^]^

The layered structure, abundant surface terminations, and redox activity of MXenes allow them to be altered in a variety of ways, when coupled with different metal ions. Metal ions (e.g., Li^+^, Na^+^, K^+^) are found to be spontaneously or electrochemically intercalated between MXene layers.^[^ ^22^ ^]^ In the case of spontaneous intercalation, delamination of the MXene and expansion of the interlayer spacing can be achieved. Controlling the interlayer spacing is generally supposed to be an effective way of changing the band gap, Fermi level, conductivity, and other properties of 2D materials. Furthermore, the intercalated metal ions hardly change the lattice structure of MXenes and still maintain high ion mobility.^[^ ^4^, ^23^, ^24^ ^]^ Electrochemical intercalation induces changes in the chemical potential and redox state of MXenes, which plays a significant role in the energy storage in metal ion batteries.^[^ ^25^, ^26^ ^]^ Interestingly, some metal ions behave differently and may induce a dramatic structural or lattice change of MXenes.^[^ ^27^ ^]^ For example, divalent metal ions (e.g., Fe^2+^, Mg^2+^) can trigger crosslinking to form a 3D network, which is beneficial for ion diffusion.^[^ ^28^, ^29^ ^]^ Some metal ions with redox activities (e.g., Fe^3+^, Cu^2+^) may generate atomic defects, pores, or produce MXene degradation. Because of the redox activities of metal ions and MXenes, MXene sheets with anchored metal atoms or nanoparticles can be synthesized in a one‐step method.^[^ ^30^, ^31^ ^]^ The evolution from MAX to MXene also involves metal ions, such as A*^x^* ^+^ oxidized from the A atoms in MAX by etchants, examples being Li^+^ in the LiF/HCl route and Cu^2+^ in the molten CuCl_2_ route. Considering that the quality of MXene sheets highly depends on the preparation process (including the etching reaction and delamination), it is vital to figure out the role of metal ions in MXene synthesis and the interactions between them.

Based on the above, it is necessary to have insight into the interactions between metal ions and MXenes. Specifically, from the perspective of MXenes, abundant surface terminations coupled with different metal ions permits effective structure control, including the interlayer structure (including interlayer spacing, surface groups, and some surface doping) and 3D structure. The modification of targeted surface terminations can be achieved by chain effects produced by metal ions, such as ‐Cl terminations produced by etching the MAX precursors in molten MCl*_x_* salts, and ‐O terminations by treating MXenes in KOH solutions.^[^ ^32^, ^33^, ^34^ ^]^ Furthermore, the transition metals in MXenes have many valence states, which provides many possible interactions with metal ions and can cause in‐plane defects, such as atomic vacancies and pores. While, from the perspective of metal ions, their interactions with MXenes can induce ion adsorption, anchoring, or in‐situ reduction, and can also form uniform active sites or nucleation sites for the production of MXene‐based metal composites. In addition, metal ions play a role as charge carriers in rechargeable batteries, and their interactions with MXenes determine the electrochemical performance. With these direct or indirect interactions, MXenes have been used for electrode materials in supercapacitors and rechargeable batteries, as excellent supports for single‐atom or hybrid catalysts, and key materials in water treatment. One of the primary targets is to develop an environmentally friendly and effective strategy for controlled MXene synthesis and the exploration of new MXenes. In this regard, insight into their interactions with metal ions helps us to understand MXene evolution from MAX precursors and may suggest ways to optimize their synthesis.

**Figure 1:**
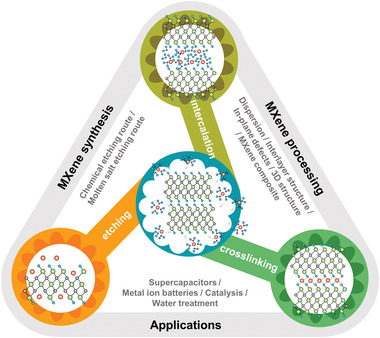
Interactions between MXene and metal ions in MXene synthesis, processing, and applications.

Over the years of research on MXenes, interactions with metal ions have been discovered and utilized, and have played crucial roles in many aspects, ranging from synthesis and processing to structure design and various applications. However, some scientific questions have not been answered, such as the different effects of metal ions on MXene structure control. Therefore, a timely review of the interaction between metal ions and MXenes is needed. This review classifies the different effects of metal ions on MXenes into three categories: intercalation, crosslinking, and etching (**Figure** **1**). The roles metal ions play in MXene synthesis, and processing are emphasized, and the ways they change its properties, structure, and surface chemistry are also discussed. The fundamental chemistry and the mechanisms of different effects or behaviors are discussed, including ion exchange, interlayer expansion, aggregation, gelation, and defect formation. Research progress on the roles metal ions have in specific cases, such as supercapacitors, metal‐ion batteries, catalysis, and water treatment, is then considered, and finally, some possible future developments are highlighted.

## 2 Different Roles of Metal Ions in MXene Synthesis and Processing

In the MXene development, metal ions play key roles in the synthesis and processing, such as MXene delamination, surface modification, structure control, property altering, MXene composite construction. The various roles are attributed to their interactions with MXenes and the unique nature of MXenes. In general, chemical interactions between guest ions and host layer solids include electrostatic interactions, charge transfer effects, *π* bonds, and strong chemical bonds. The match of their energy levels is the key for the interaction, which is associated with the chemical structure and surface chemistry of the host.^[^ ^27^ ^]^ For graphite‐like materials with non‐polar surface and delocalization *π* electrons, both electron acceptors and donors are allowed to create interactions by charge transfer effects, and electrons flow away from or into the their conduction band. TMDs‐like materials only accept electrons to fill the d‐orbitals, and thus electron acceptors do not create interactions. However, clays have fundamentally different behaviors because of their surface functional groups. Electrostatic interactions and coordination interactions with metal cations are the primary forms. For MXene materials, it is well known that they have abundant surface terminations (‐O/‐OH/‐F/‐Cl) like clays, and thus expectedly have spontaneous ion intercalation or exchange behavior due to electrostatic interactions. In addition, MXenes are composed of transition metal atoms like TMDs, so that their various valence states guarantee the possibility of charge transfer or oxidation/reduction reactions. In this case, three main effects of metal ions on MXenes are classified, including intercalation, crosslinking and etching. The roles in the MXene synthesis and processing are introduced, and the corresponding fundamental interaction chemistry is also discussed.

### 2.1 Metal Ion Intercalation

#### 2.1.1 Intercalation in MXene Synthesis

Unlike van der Waals forces between the graphenes in graphite, there are metallic M‐A bonds between the MXene layers in the MAX phase, which are hard to break by mechanical forces. Therefore, chemical (electro‐chemical) reactions are necessary, and oxidizing etchants that react with the A layers in MAX are generally used.^[^ ^11^, ^35^ ^]^ Additional intercalation is also required to obtain single or few‐layer MXene sheets. The first report of the synthesis of the MXene, Ti_3_C_2_T*_x_*, was in 2011, where it was produced by immersing a Ti_3_AlC_2_ precursor in a HF solution, where the selective etching of Al layers resulted in a graphene‐like structure, and certain terminations (‐O, ‐OH, and/or ‐F) were introduced on the surface.^[^ ^7^ ^]^ The obtained MXene (HF‐MXene) could be spontaneously intercalated with organic molecules (e.g., dimethyl sulfoxide, urea) and cations (e.g., Li^+^, Na^+^, K^+^).^[^ ^22^, ^36^ ^]^ Given this post‐intercalation ability, a one‐step and milder synthesis route was proposed in 2014, using a mixture of hydrochloric acid (HCl) and lithium fluoride salt (LiF) as the etchant. Compared with HF‐MXene, a dramatic change in the c‐lattice parameter (c‐LP, including the thickness of two MXene flakes and their interlayer spacing) with Li^+^ intercalation was observed, causing easier delamination and fewer defects.^[^ ^37^ ^]^

**Figure 2:**
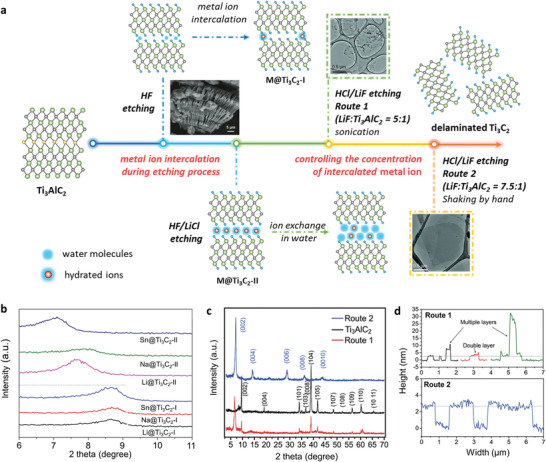
The effect of metal ion intercalation in MXene synthesis. a) Schematic of metal ion intercalation in MXene synthesis. Route 1: the molar ratio of LiF and Ti_3_AlC_2_ is 5:1; Route 2: the molar ratio of LiF and Ti_3_AlC_2_ is 7.5:1. The inset SEM image is multilayer Ti_3_C_2_T*_x_* prepared by HF etching. The inset TEM images are Ti_3_C_2_T*_x_* flakes produced by Route 1 and 2. Left‐hand side: Reproduced with permission.^[^ ^7^ ^]^ Copyright 2011, Wiley‐VCH. Right‐hand side: Reproduced with permission.^[^ ^40^ ^]^ Copyright 2016, Wiley‐VCH. b) XRD patterns of M@Ti_3_C_2_‐I and M@Ti_3_C_2_‐II. Reproduced with permission.^[^ ^39^ ^]^ Copyright 2020, Elsevier. c) XRD patterns of Ti_3_C_2_T*_x_* MXene prepared by Route1 and Route 2. d) AFM height profiles of Ti_3_C_2_T*_x_* flakes produced by Route 1 and 2. Those produced by Route 2 have the same height of ≈2.7 nm and are identified as monolayers. Reproduced with permission.^[^ ^40^ ^]^ Copyright 2016, Wiley‐VCH.

It has been shown that the cation intercalation during etching process plays a vital role in the synthesis, in addition to the formation of HF. As illustrated in **Figure** **2**a, H_2_O molecules occupy some of the Ti_3_C_2_ interlayer space, after the removal of Al atoms by the HF solution. Only a small number of cations (M*^x^* ^+^) can be intercalated with an uneven distribution (M@Ti_3_C_2_‐I, Figure 2a), and thus a small structural change is produced. In contrast, if the etching and M*^x^* ^+^ ion intercalation are carried out simultaneously, the hydrated cations are evenly distributed between the layers, resulting in a larger interlayer spacing which facilitates exfoliation (M@Ti_3_C_2_‐II, Figure 2a).^[^ ^38^, ^39^ ^]^ This is shown by the XRD patterns in Figure 2b. When HF‐Ti_3_C_2_ is intercalated with LiCl, NaCl or SnCl_4_·5H_2_O (i.e., Li@Ti_3_C_2_‐I, Na@Ti_3_C_2_‐I, or Sn@Ti_3_C_2_‐I), there is only a slight shift of the (0002) peak from its original position at 8.91°. However, when HF/LiCl, HF/NaCl or HF/SnCl_4_ is the etchant in MXene synthesis, a larger peak shift is observed for M@Ti_3_C_2_‐II (i.e., Li@Ti_3_C_2_‐II, Na@Ti_3_C_2_‐II, or Sn@Ti_3_C_2_‐II). The c‐LP value of the obtained MXene is increased from 20.32 Å (Li@Ti_3_C_2_‐I) to 23.00 Å (Li@Ti_3_C_2_‐II) by Li^+^ intercalation, and from 20.44 Å (Sn@Ti_3_C_2_‐I) to 24.84 Å (Sn@Ti_3_C_2_‐II) by Sn^4+^ intercalation. Moreover, the MXene obtained by in situ intercalation during the etching process (i.e., M@Ti_3_C_2_‐II) involves ion exchange and swelling, which makes the MXene delamination easier.

Large MXene flakes of high quality can also be obtained by controlling the concentration of intercalated metal ions.^[^ ^40^ ^]^ Two different synthesis routes with different molar ratios of LiF and Ti_3_AlC_2_ are shown in Figure 2a. In Route 1, the molar ratio of LiF to Ti_3_AlC_2_ is 5:1, and the LiF reacts with HCl to produce HF in etching process. Comparing with Route 1, Route 2 provides excess Li^+^ for the intercalation, and twice the amount of HCl is used to satisfy the Al etching. The combined effect improves the reaction kinetics resulting in the complete conversion of Ti_3_AlC_2_ to Ti_3_C_2_T*_x_* (Figure 2c) and accelerates the H_2_O uptake during washing to facilitate delamination. Thus, shaking by hand is enough to obtain monolayer MXene, instead of sonication or additional delamination. The obtained flakes have large lateral dimensions (4–15 µm) and a uniform thickness (≈2.7 nm), as shown in Figure 2d. Compared with the calculated thickness of a single layer (0.98 nm),^[^ ^41^ ^]^ the increased thickness measured by AFM is due to surface adsorbates, such as water molecules. Moreover, the MXene flakes produced using Route 2 show well‐defined and clean edges with a much lower defect concentration in high‐resolution TEM images, resulting in better electronic properties.

#### 2.1.2 Intercalation in MXene Processing

**Figure 3:**
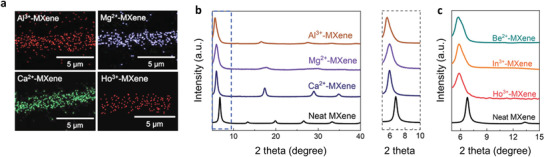
The effect of metal ion intercalation in MXene processing. a) EDS element mapping of Al^3+^‐MXene, Mg^2+^‐MXene, Ca^2+^‐MXene, and Ho^3+^‐MXene. The MXene here is Ti_3_C_2_T*_x_*. b) XRD patterns of neat MXene, Ca^2+^‐MXene, Mg^2+^‐MXene, and Al^3+^‐MXene under 75% relative humidity at room temperature. c) XRD patterns of Be^2+^‐MXene, In^3+^‐MXene, Ho^3+^‐MXene, and neat MXene. Metal ion intercalation enlarges the interlayer spacing. Reproduced with permission.^[^ ^42^ ^]^ Copyright 2020, Wiley‐VCH.

It is well known that intercalation is an effective way to alter the physical and chemical properties of 2D materials, including the band gap, conductivity, optical and mechanical properties, chemical reactivity, etc. A larger interlayer spacing increases ion diffusion and exposes more active sites, significantly improving the electrochemical performance.^[^ ^4^ ^]^ MXenes prepared by the LiF/HCl or HF/LiCl method can be intercalated with various other metal cations, including univalent ions (e.g., Na^+^, K^+^) and multivalent ions (e.g., Be^2+^, Ca^2+^, Mg^2+^, Zn^2+^, Mn^2+^, In^3+^, Ho^3+^, Al^3+^).^[^ ^42^, ^43^ ^]^ After immersing the MXenes in different salt solutions, intercalants remain in the interlayer space due to electrostatic interactions (**Figure** **3**a) and therefore increase it. The interlayer expansion is different for different metal ions. Li et al. prepared metal ion (M^n+^) intercalated Ti_3_C_2_T*_x_* MXene electrodes (M^n+^‐MXene) with controllable interlayer spacings. As shown in Figure 3b,c, the (0002) peak shifted to a lower angle, from 6.7° (neat MXene produced by LiF/HCl etching) to 5.9° (Ca^2+^‐MXene), 5.9° (Mg^2+^‐MXene), 5.6° (Al^3+^‐MXene), 5.9° (Ho^3+^‐MXene), 5.8° (In^3+^‐MXene), and 5.7° (Be^2+^‐MXene), corresponding to expanded d‐spacings (equal to c‐LP/2) of 13.2, 14.7, 15.0, 15.6, 15.3, 15.2, and 15.1 Å, respectively. The change in d‐spacing is explained by the intercalation chemistry and the related hydrated radius of metal ion, which will be explained in the next section. As expected, the M^n+^‐MXene electrodes have an almost negligible interfacial resistance with the electrolyte, indicating that the charge‐transfer and ion diffusion are increased by the presence of the intercalants.

Apart from the interlayer spacing, the mechanical properties, electrical conductivity, and stability of MXenes are also influenced.^[^ ^27^, ^44^, ^45^ ^]^ The introduction of intercalants (e.g., Li^+^, dimethylsulfoxide, and tetraalkylammonium hydroxide) is thought to result in a lower conductivity, worse mechanical properties, and instability.^[^ ^27^, ^46^ ^]^ Removing the intercalants by cation exchange in an acid solution^[^ ^46^ ^]^ or annealing^[^ ^47^ ^]^ can significantly increase the electrical conductivity. However, Osti et al. found that K^+^ intercalation improved stability and structural homogeneity, and decreased the mobility of water confined between layers.^[^ ^48^ ^]^ It was also shown by Ding et al. that the intercalation of Al^3+^ ions improved the stability of a Ti_3_C_2_T*_x_* membrane in aqueous solution and there was no structural collapse after long‐time soaking in water.^[^ ^49^ ^]^ An increase in the tensile strength of a Ti_3_C_2_T*_x_* film produced by Al^3+^ intercalation was reported by Liu et al., while the intrinsic electrical conductivity was maintained.^[^ ^50^ ^]^ Therefore, more research is needed to clarify the effects of metal ion intercalation on the mechanical properties, electrical conductivity, and stability of MXenes.

The effect of intercalation on the electronic structure and surface chemistry has also been also investigated. Using X‐ray absorption spectroscopy (XAS), Al‐Temimy et al. found that cation intercalation (Li^+^, Na^+^, K^+^, and Mg^2+^) strongly affected the oxidation state of the Ti atoms in Ti_3_C_2_T*_x_* MXene after drying in air, while H_2_SO_4_ pre‐treatment prevented it.^[^ ^51^ ^]^ Combining XAS with X‐ray photoemission electron microscopy (X‐PEEM), the Ti oxidation states of Li‐, and Mg‐Ti_3_C_2_T*_x_* were found to depend on their thickness, while those of Na‐ and K‐Ti_3_C_2_T*_x_* did not.^[^ ^52^ ^]^ This difference is related to many factors, such as the probing depth, the particle geometry, and the amount of water confined in the MXene layers under different pressures. Overall, the impact of the intercalation of different metal ions on MXene properties differs and this requires further investigation. Furthermore, metal cation intercalation in alkali solutions may lead to a larger d‐spacing^[^ ^22^ ^]^ than in neutral solutions and to a change in surface terminations.^[^ ^53^ ^]^ Halim et al. showed that ‐F terminations (from 1.0 to 0.7 moles per mole of Ti_3_C_2_T*_x_*) produced by fluoride etchants during MXene synthesis were replaced by ‐O terminations (from 0.5 to 1.2 moles; ‐OH, from 0.4 to 0.5 moles per mole of Ti_3_C_2_T*_x_*) after NaOH treatment.^[^ ^54^ ^]^

#### 2.1.3 The Intercalation Mechanism

As mentioned above, metal ion intercalation significantly weakens the interaction between layers, which helps overcome the sluggish exfoliation kinetics during the synthesis and alters the physical and chemical properties of MXenes. During the initial Ti_3_C_2_ exfoliation step in a HF solution, the strong bonding between Ti and F atoms weakens the Ti‐Al bonds, which permits HF molecular intercalation into Ti_3_AlC_2_ to etch Al atoms by forming AlF_3_ and H_2_, accompanied by Ti‐F bond formation. With the Ti_3_C_2_ layer isolation, H_2_O molecules penetrate the interlayer space to support the exfoliation process by delaminating the Ti_3_C_2_T*_x_* and forming ‐OH/O terminations the resulting surfaces.^[^ ^55^ ^]^ In most cases, the H_2_O intercalation is promoted by the hydration of metal ions due to the strong electrostatic interactions between the negatively charged Ti_3_C_2_T*_x_* surface and metal cations, in contrast with the considerably weaker interaction with pure H_2_O.^[^ ^27^ ^]^ The zeta potential of the Ti_3_C_2_T*_x_* dispersion is lower than −30 mV over a wide pH range,^[^ ^56^, ^57^ ^]^ which explains why the sluggish exfoliation kinetics is hastened by metal ion intercalation in the HF/Li and LiF/HCl methods.

**Figure 4:**
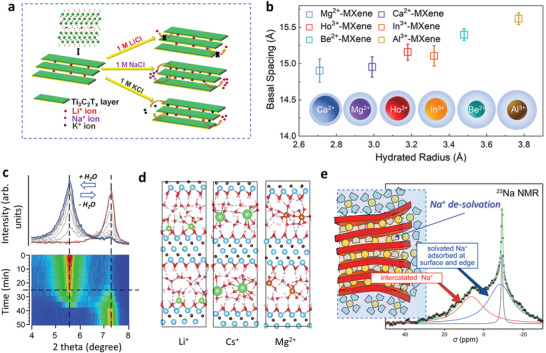
The mechanism of metal ion intercalation. a) Illustration of ion exchange in a clay‐like MXene. The ion exchange is generally thought to occur between the solution and ions located between the MXene layers, accompanied by H^+^ release. Reproduced with permission.^[^ ^58^ ^]^ Copyright 2017, American Chemical Society. b) Correlation of the interlayer spacing of M*^x^* ^+^‐MXene (including Ca^2+^, Mg^2+^, Ho^3+^, In^3+^, Be^2+^, and Al^3+^) electrodes and the hydrated radius of intercalated metal ions (obtained from Marcus's group). Reproduced with permission.^[^ ^42^ ^]^ Copyright 2020, Wiley‐VCH. c) XRD patterns of ion‐intercalated Ti_3_C_2_T*_x_* MXene with humidity control. Reproduced with permission.^[^ ^38^ ^]^ Copyright 2016, American Chemical Society. d) Li^+^, Cs^+^ and Mg^2+^ representative atomic arrangements around different cations confined in two Ti_3_C_2_T*_x_* layers. Reproduced with permission.^[^ ^60^ ^]^ Copyright 2020, Royal Society of Chemistry. e) ^23^Na NMR spectrum to investigate the mechanism of Na^+^ intercalation into Ti_3_C_2_T*_x_*, during electrochemical cycling. Reproduced with permission.^[^ ^67^ ^]^ Copyright 2016, American Chemical Society.

The mechanism of the intercalation of various metal ions by immersing MXene in different salt solutions has also been investigated. Sharma et al. found by ICP‐MS measurements that the clay‐like (Li^+^‐intercalated) Ti_3_C_2_T*_x_* released Li^+^ and H^+^ ions and took up K^+^ and Na^+^ ions, when immersed in 1 m KCl and 1 m NaCl, respectively.^[^ ^58^ ^]^ However, no ion exchange occurred in 1 m LiCl, with almost zero driving force (**Figure** **4**a). The ion exchange process is complex, and is controlled by kinetic and thermodynamic factors. With immersion calorimetry, the K^+^‐Li^+^ exchange and Li^+^ de‐intercalation are exothermic, while Na^+^‐Li^+^ exchange is endothermic. The greater extent of the Na^+^‐Li^+^ exchange is due to the higher ion mobility. The effect of the intercalation of different ions has been addressed, especially the effect on interlayer spacing as shown in Figure 3b, which is supposed to be related to the hydration state of the cations. A larger d‐spacing is caused by the intercalated metal ion of a larger hydrated radius (Figure 4b). Moreover, the interlayer spacing is found to change with the humidity level on account of the H_2_O uptake or loss. Using in situ XRD and thermogravimetric analysis, Ghidiu et al. suggested that the expansion was related to the hydration enthalpy of the cations (Figure 4c).^[^ ^38^ ^]^ A cation with a higher charge/radius ratio tends to have a higher absolute value of hydration enthalpy,^[^ ^59^ ^]^ suggesting a tendency to form a bilayer H_2_O structure between layers at lower humidity. Thus, Li^+^ and Na^+^‐MXene start to expand at ≈90% relative humidity (RH), while Mg^2+^‐MXene responds at ≈10% RH.

The specific intercalation states and interfacial chemistry of different metal ions between two MXene sheets have been tracked, which helps provide a deeper understanding of MXene energy storage properties and processes. Gao et al. used ab initio molecular dynamics (AIMD) simulations to investigate the atomic arrangements around Li^+^, Na^+^, K^+^, Cs^+^, and Mg^2+^ confined in two Ti_3_C_2_T*_x_* MXene layers (Figure 4d).^[^ ^60^ ^]^ As previously reported, there was a certain number of water molecules around each cation, while the hydrated cations had different locations between the MXene layers. Li^+^, Na^+^, and K^+^ prefer to be on the surface, while Cs^+^ and Mg^2+^ prefer to be between the layers. Radial distribution functions (RDF) quantified the relative position between ‐O surface termination (O_T_) and cations. The capacitance at the open circuit potential (OCP) was measured to be in inverse proportion to this cation‐O_T_ distance. Considering the effect of water molecules confined between MXene layers, a modified electric double layer (EDL) model was proposed to explain this relationship. At the same time, the EDL energy storage mechanism was identified in the neutral aqueous electrolytes, which will be discussed later in detail.

Understanding intercalation chemistry in a nonaqueous system is also essential, especially during the electrochemical process. Metal cations, also the charge carriers, intercalate and de‐intercalate the MXene layers to achieve energy storage and release, and the state of the intercalated ions needs to be understood.^[^ ^61^, ^62^, ^63^ ^]^ Kajiyama et al. used solid‐state ^23^Na NMR to reveal the Na^+^ intercalation mechanism. As presented in Figure 4e, 3 different Na^+^ signals can be detected, representing solvated Na^+^ from the residual electrolyte, solvated Na^+^ trapped in the SEI layer or adsorbed on the surface, and completely or partially de‐solvated Na^+^ intercalation. It is believed that de‐solvated Na^+^ intercalation is the main contributor to energy storage. Furthermore, the solvent effect has also been investigated. Wang et al. demonstrated Li^+^ intercalation with a complete de‐solvation behavior in a propylene carbonate (PC) system. However, solvent co‐intercalation occurs in acetonitrile (ACN) and dimethylsulfoxide (DMSO) systems.^[^ ^64^ ^]^ Remarkably, the efficient de‐solvation in PC results in a high volumetric capability. The interaction between intercalated ions and the MXene surface is supposed to determine the electrochemical behavior of MXenes during charging.^[^ ^65^, ^66^ ^]^ Regretfully, few studies have focused on intercalation chemistry in a nonaqueous system, and more attention needs to be paid to this subject.

### 2.2 Metal Ions as Crosslinking Agents

#### 2.2.1 Crosslinking in MXene Processing

**Figure 5:**
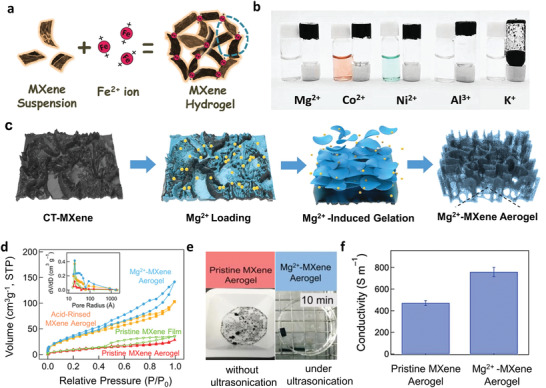
Metal ions as crosslinking agents to initiate the gelation of Ti_3_C_2_T*_x_* MXene. a) Schematic of Ti_3_C_2_T*_x_* MXene hydrogel formation initiated by Fe^2+^ ion interaction. b) Photos of a Ti_3_C_2_T*_x_* MXene hydrogel with crosslinking agents of Mg^2+^, Co^2+^, Ni^2+^, Al^3+^ and K^+^. Reproduced with permission.^[^ ^28^ ^]^ Copyright 2019, Wiley‐VCH. c) Schematic of Mg^2+^‐MXene aerogels produced from the CT‐MXene platform and Mg^2+^‐induced gelation. d) N_2_ adsorption‐desorption isotherms of the original MXene film, the Mg^2+^‐MXene aerogel, and the MXene aerogel after acid washing. The inset Figure in (d) shows the corresponding pore size distributions. e) Photos of a MXene aerogel (without Mg^2+^ intercalation) and an Mg^2+^‐MXene aerogel after ultrasonication for 10 min. f) Electrical conductivities of pristine MXene and Mg^2+^‐MXene aerogels. Reproduced with permission.^[^ ^68^ ^]^ Copyright 2021, Wiley‐VCH.

The addition of a metal salt solution can alter the stability of Ti_3_C_2_T_x_ colloidal suspensions, and flocculation or gelation occurs due to the destruction of electrostatic repulsive forces between the sheets. The treatment with alkali metal hydroxides (e.g., NaOH, KOH) can result in Ti_3_C_2_T*_x_* nanosheet aggregation and a crumpled structure, with the intercalation of metal ions.^[^ ^28^, ^68^, ^69^, ^70^ ^]^ A 3D nanoribbon structure can be fabricated using an increased alkali concentration.^[^ ^71^, ^72^, ^73^ ^]^ A high concentration NaCl solution was also found to induce a crumpled morphology. Interestingly, some multivalent metal ions (e.g., Fe^2+^, Mg^2+^, Co^2+^, Ni^2+^, Mg^2+^, Al^3+^) induce Ti_3_C_2_T*_x_* nanosheet crosslinking to form a stable 3D hydrogel, rather than simple flocculation or aggregation (**Figure** **5**a,b).^[^ ^28^ ^]^ The gelation process is accomplished in minutes when the MXene concentration is high enough (≥ 6 mg mL^−1^). The MXene concentration is a key factor, as only flocculation occurs in low concentration, because of the limited interaction range and fast charge neutralization of metal ions. Following this, Ding et al. fabricated a 3D Ti_3_C_2_T*_x_* MXene aerogel by Mg^2+^ crosslinking, without polymeric binders or templates.^[^ ^68^ ^]^ Using the thermal contraction of polystyrene (PS) substrate, a crumple‐textured Ti_3_C_2_T*_x_* coating (CT‐MXene) was pre‐prepared. This CT‐MXene allows a higher Mg^2+^ loading, and which induces gelation on the surface after soaking in a Ti_3_C_2_T*_x_* dispersion (21 mg mL^−1^), as illustrated in Figure 5c. The (0002) peak shift indicates that the Mg^2+^ ions are intercalated. The aerogel formed is really tough because of the strong crosslinking, so that the pore structure is preserved even after the intercalated Mg^2+^ is removed by acid rinsing with only a slight change in specific surface areas (from 140.5 to 131.0 m^2^ g^−1^, Figure 5d). The aerogel remained intact under ultrasonication in water, in contrast to the quick destruction of the aerogel without Mg^2+^ crosslinking (Figure 5e). The strong interaction also ensures better inter‐sheet connections and uninterrupted electron diffusion paths, indicated by the aerogel having a higher electronic conductivity (758.4 ± 41.6 S m^−1^) than the original (471.3 ± 22.8 S m^−1^, Figure 5f). Lin et al. fabricated highly stable Ti_3_C_2_T*_x_* foams by placing the metal substrate (Zn, Al, Fe, Co, and Ni) to an acidic MXene or MXene/GO hybrid dispersion, where the metal ions diffuse out and induce gelation.^[^ ^74^ ^]^ Large pore size and thick lamellas tend to form in a higher ion concentration and a quicker assembly process by pH control. The prepared foams can be loaded with different nanoparticles (SiO_2_ and Fe_3_O_4_).

#### 2.2.2 The Crosslinking Mechanism

Before introducing the crosslinking behavior of metal ions, the aggregation of MXene flakes in alkali metal hydroxides and salt solutions of high concentration will be explained first.^[^ ^75^, ^76^, ^77^ ^]^ The main reason is the imbalance between electrostatic attraction and electrostatic repulsion.^[^ ^69^, ^78^ ^]^ The OH^−^ in alkali metal hydroxides (e.g., NaOH) passivate the positively charged edge sites of Ti_3_C_2_T*_x_* flakes, while Na^+^ is adsorbed on the negatively charged surface sites. The positive Na^+^ ions attract the adjacent MXene nanosheets, which results in a large number of face‐to‐face interactions between them. In the case of a salt solution, the Debye length decreases with the increased salt concentration, which indicates that the repulsion between the nanosheets is reduced, creating a face‐to‐face interaction between them.^[^ ^79^ ^]^ This strong interaction causes the nanosheet aggregation and a crumpled structure after drying, which may also account for the nanoribbons formed in high concentration alkali metal hydroxides, as mentioned earlier. The high concentration of OH^−^ may over‐passivate the edge making it negatively charged. Meanwhile, the increased concentration of Na^+^, which prefers to be absorbed on the surface, attracts the negatively charged edge. Such a plane face‐to‐edge attraction induces the high‐degree of folding and crumping of the MXene sheet, and thus nanoribbons are formed. This process is like rolling a sheet of paper into a tube‐shape from one edge side.

**Figure 6:**
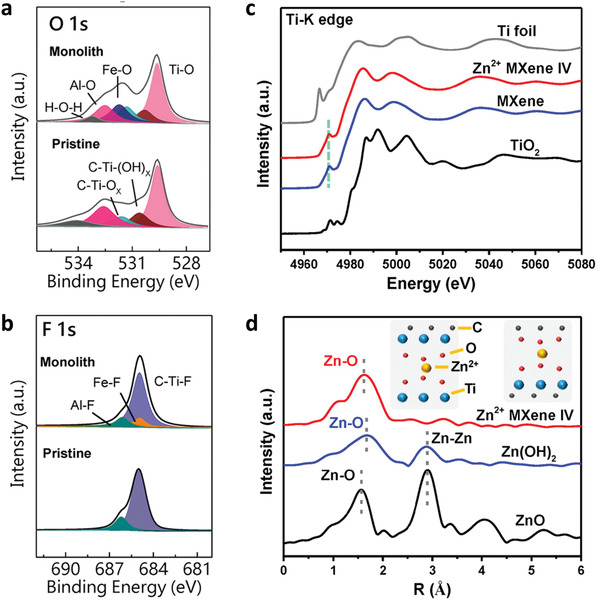
The mechanism of ion crosslinking. a,b) O *1s* and F *1s* XPS spectra of pristine Ti_3_C_2_T*_x_* MXene and a MXene monolith. Reproduced with permission.^[^ ^28^ ^]^ Copyright 2019, Wiley‐VCH. c) The normalized XANES at the Ti K‐edge of Zn^2+^ Ti_3_C_2_T*_x_* MXene foam. d) Fourier transform extended X‐ray absorption fine structure spectra of Zn^2+^ MXene foam. The gelation process did not cause obvious degradation of the MXene sheets, and the Zn^2+^ ionic state of the Zn sites remained. Reproduced with permission.^[^ ^74^ ^]^ Copyright 2020, American Chemical Society.

Compared with aggregation and intercalation, the direct chemical or ionic bonding between multivalent metal ions (e.g., Fe^2+^, Mg^2+^, Co^2+^, Ni^2+^, Mg^2+^, Al^3+^) and surface terminations is greater in the crosslinking. Giving the Ti_3_C_2_T*_x_* gelation process with Fe^2+^ ions as an example. Firstly, the electrostatic interactions with Fe^2+^ ions and negatively charged Ti_3_C_2_T*_x_* sheets break the original electrostatic balance among sheets. Then, as linkers, the ions interact with ‐OH/‐O and ‐F groups on MXene surface.^[^ ^28^ ^]^ In this process, the hydration shells around Fe^2+^ are destroyed, as obvious Fe‐O and Fe‐F bonds (very low intensity) are detected in in XPS spectra (**Figure** **6**a,b). What is more, the increase in interlayer spacing is only ≈0.12 nm, close to the bare ionic radius of Fe^2+^ without the hydration shell.^[^ ^74^ ^]^ The crosslinking mechanism with Zn^2+^ ions is also revealed by XAS, where the electronic structure and local environments of Ti and Zn is investigated.^[^ ^74^ ^]^ The normalized Ti K‐edge XANES spectra in Figure 6c indicated that no obvious MXene degradation occurred, as the signal of Zn^2+^‐MXene resembled that the original MXene but differed from that of TiO_2_. The Zn K‐edge XANES spectrum of Zn^2+^‐MXene is similar to that of ZnSO_4_, indicating the zinc is in the Zn^2+^ ionic state. Moreover, Zn‐O complexes with an atomic dispersion on MXene foam are the primary interaction form, as exhibited in Figure 6d, and XPS results confirm the Zn‐O ionic bonding. The above results show that the strong electrostatic interactions between multivalent metal ions and negatively charged terminations of adjacent MXene flakes break the shielding effect of hydration layer, which allow the ionic bonding to form. Meanwhile, the high concentration of MXene sheets provides more interaction chances and helps the formation of crosslinking network. However, the research on the crosslinking mechanism is still scarce until now, and more investigations are required, such as the effect of surface groups and MXene species.

### 2.3 The Etching Effect of Metal Ions

#### 2.3.1 Etching Effect in MXene Synthesis

The molten salt route is becoming more important in MXene synthesis, in addition to the wet chemical etching route. In this approach, the metal ions (e.g., Cu^2+^, Zn^2+^, Cd^2+^, Co^2+^) act as the oxidant to etch the A layer (e.g., Si, Al) in the MAX phase.^[^ ^12^, ^13^, ^14^, ^80^ ^]^ For example, Ti_3_SiC_2_ reacts with molten CuCl_2_ at 750 °C and produces Ti_3_C_2_Cl_2_, Cu clusters, and SiCl_4_ gas. By coupling different MAX phases with appropriate molten salts (including cations and anions), a broad family of MXene materials can be theoretically synthesized, with different surface chemistries and structures. Similar to the HF route, Ti vacancies are also found, which indicates that the metal ions not only etch A atoms but also Ti atoms. Because of this, it is essential to study the interaction between metal ions and MXene during synthesis in molten salts.

**Figure 7:**
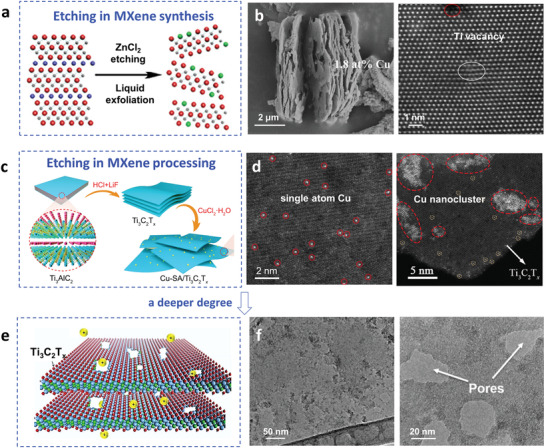
The etching of metal ions in MXene during Ti_3_C_2_T*_x_* MXene synthesis and processing. a) Schematic of Ti vacancies generated in the synthesis of Ti_3_C_2_T*_x_* MXene in molten ZnCl_2_ salts. At the same time, single zinc atoms are produced and immobilized on the MXene layers (Zn‐MXene). Reproduced with permission.^[^ ^81^ ^]^ Copyright 2020, American Chemical Society. b) SEM image and HAADF‐STEM image of a single atom Cu catalyst synthesized by etching Ti_3_AlNC in molten CuCl_2_. Reproduced with permission.^[^ ^82^ ^]^ Copyright 2021, Elsevier. c) Schematic of the Cu‐SA/Ti_3_C_2_T*_x_* synthesis procedure, by adding a certain amount of CuCl_2_·2H_2_O to a Ti_3_C_2_T*_x_* suspension. (d) HAADF‐STEM images of Cu‐SA/Ti_3_C_2_T*_x_* and Cu‐NC/Ti_3_C_2_T*_x_*. Here, 0.67 mL CuCl_2_·2H_2_O (1 mg mL^–1^) for Cu‐SA/Ti_3_C_2_T*_x_* and 2.01 mL for Cu‐NC/Ti_3_C_2_T*_x_* were used. Reproduced with permission.^[^ ^84^ ^]^ Copyright 2021, Springer Nature. e) Diagram of the porous Ti_3_C_2_T*_x_* MXene structure produced by the chemical etching of Cu^2+^ ions. f) High‐resolution TEM images of a Ti_3_C_2_T*_x_* flake after soaking in a 0.2 m CuSO_4_ solution at RT. The left image is before acid washing and shows TiO_2_ nanoparticles on the surface, and the right one is after acid washing showing pores. Reproduced with permission.^[^ ^85^ ^]^ Copyright 2016, Wiley‐VCH.

It has been reported by Gu et al. that specific numbers of Ti vacancies are introduced by reacting Ti_3_AlC_2_ with ZnCl_2_ at 550 °C for 10 h, and, at the same time, Zn atoms are immobilized on the MXene layer (**Figure** **7**a).^[^ ^81^ ^]^ Similarly, Song et al. introduced Ti defects by etching Ti_2_AlN in molten CuCl_2_ (the molar ratio of MAX to salt was 1:2) at 1000 °C for 7 h, or in molten CoCl_2_ (800 °C, 7 h).^[^ ^82^ ^]^ After the reaction, the products were treated with a HCl solution and FeCl_3_ to remove Cu particles, or only a HCl solution to remove Co particles. The final products had a visible layered structure with a Cu content of 1.8 at% (Figure 7b). A Ti vacancy marked by a red circle in the HAADF‐STEM images was observed, and the lattice mismatch of Ti atoms is indicated by the white ellipse. Cu single atoms with a valence state between 0 and +2 were also detected, as indicated in the XANES results. In addition to the Ti vacancies and anchoring of single atom metals, in‐plane pores were also generated by etching the MAX precursor in molten salts. Zhang et al. found that Ti_3_C_2_Cl_2_ with in‐plane pores was synthesized by reacting Ti_3_AlC_2_ with a NaCl/ZnCl_2_ salt mixture at 550 °C for 5 h.^[^ ^83^ ^]^ With an increasing mole fraction of NaCl, the micropores gradually evolved into mesopores (3–4 nm), and a specific surface area of 85 m^2^ g^−1^ was achieved.

#### 2.3.2 Etching Effect in MXene Processing

For MXenes prepared by wet chemical etching, similar effects can be achieved by post‐processing with salt solutions. As illustrated in Figure 7c, single copper atoms can be anchored on Ti_3_C_2_T*_x_* sheets by slowly adding a certain amount of CuCl_2_·2H_2_O (1 mg mL^–1^; 0.67 mL) to a Ti_3_C_2_T*_x_* dispersion.^[^ ^84^ ^]^ No Cu peak was detected in the XRD pattern. The valence state of the Cu atom is calculated to be +0.42 from the XAS results, and uniform Cu single atoms on the Ti_3_C_2_T*_x_* lattice are observed as bright dots (Figure 7d). Cu single atoms and Cu nanoclusters formed when treated with a larger amount of CuCl_2_·2H_2_O (2.01 mL), are shown in the HAADF‐STEM image (Figure 7d). The coordination conditions of the Cu are Cu‐O and Cu‐Cu, from the FT‐EXAFS curves. Furthermore, by increasing the Cu^2+^ concentration, Ti_3_C_2_T_x_ degradation and in‐plane etching occurred, as shown in Figure 7e.^[^ ^85^, ^86^ ^]^ Porous Ti_3_C_2_T*_x_* flakes (pore size: ≈10 nm) were fabricated by Ren et al. by mixing a delaminated Ti_3_C_2_T*_x_* colloidal solution with a CuSO_4_ solution, followed by acid washing (Figure 7f). During the process, the flakes were partially oxidized to TiO_2_ which was removed by diluted HF acid (5 wt%). Not only Cu^2+^ but many other metal ions such as Co^2+^ and Fe^3+^ have been reported to have etching ability. By controlling the concentration of Cu^2+^, the pore size and density could also be changed. Xiong et al. found that a CuSO_4_ concentration of 0.2 M generated pores ranging in diameter from 2 to 15 nm by later HF acid (5 wt%) treatment.^[^ ^87^ ^]^ A slightly higher concentration (0.4 M) produced a similar pore size but a higher pore density. When the concentration was increased to 1 m, the pore size increased, ranging from 15 to 25 nm.

Some other metal ions (e.g., Cd^2+^, Ru^3+^, Ag^+^, AuCl_4_^−^, PdCl_4_^−^)^[^ ^88^, ^89^, ^90^, ^91^, ^92^ ^]^ are also self‐reduced to lower or zero valence states when in contact with MXenes, in the absence of extra reagents. Peng et al. fabricated Ru‐doped Mo_2_CT*_x_* sheets by adding RuCl_3_ to the MXene dispersion.^[^ ^91^ ^]^ It is thought that the Mo vacancies produced in the synthesis from Mo_2_GaC initiate the reduction of Ru^3+^ ions, accompanied by an increased oxidation state of the nearby Mo atoms. XAS and XPS results indicate that Ru atoms are introduced into the Mo_2_CT_x_ lattice and occupy the Mo sites without Mo‐Ru bonding. In addition, a higher dose of RuCl_3_ leads to Ru nanoparticles forming on the surface. Satheeshkumar et al. directly deposited Ag, Au, and Pd nanoparticles onto the Ti_3_C_2_T*_x_* surface, by adding the corresponding salts (AgNO_3_, HAuCl_4_, and PdCl_2_) to a MXene suspension.^[^ ^93^ ^]^ Peaks of Ag, Au, and Pd metals were detected in the XRD patterns. It was also reported by Xie et al. that visible metal layers formed on the surface after the MXene film was treated with water containing Ag^+^ or AuCl_4_^−^ ions.^[^ ^89^ ^]^

#### 2.3.3 The Etching Mechanism

**Figure 8:**
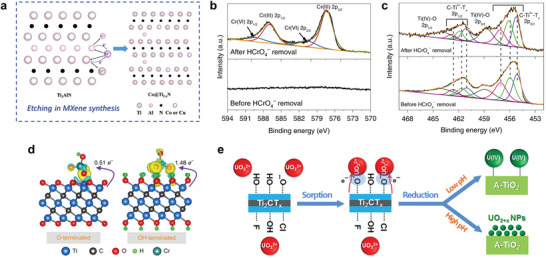
The mechanism of metal ion etching. a) A diagram of the anchoring of single metal atoms on Ti vacancies during molten salt etching. Reproduced with permission.^[^ ^82^ ^]^ Copyright 2021, Elsevier. b,c) High‐resolution Cr *2p* and Ti *2p* XPS spectra of Ti_3_C_2_T*_x_*‐based films before and after HCrO_4_^−^ removal. The valence state of Cr in HCrO_4_^−^ is reduced from Cr(VI) to Cr(III), accompanied by a valence state increase of Ti in Ti_3_C_2_T*_x_*. d) Differences in charge density of HCrO_4_^−^ on O‐ and OH‐terminated Ti_3_C_2_. The turquoise and yellow regions indicate depletion and accumulation of electrons, respectively. Reproduced with permission.^[^ ^89^ ^]^ Copyright 2019, Springer Nature. e) Schematic illustration of U(VI) removal at different pH values. At low pH, the reduced U(IV) species is identified as mononuclear with bidentate binding to the MXene substrate. At near‐neutral or high pH, nanoparticles of the UO_2+_ *_x_* phase adsorb on the substrate with some Ti_2_CT*_x_* being transformed to amorphous TiO_2_. Reproduced with permission.^[^ ^88^ ^]^ Copyright 2018, American Chemical Society.

The interaction between metal ions and MXenes can generate in‐plane defects (vacancies and pores) and may even degrade the MXenes. Simultaneously, the valence state of the metal ions is reduced, and even zero valent metals (e.g., Ag, Au, Pt) can be directly produced directly, as mentioned earlier. For the mechanism of MAX etching in molten salts, it is widely believed that the A element prefers to be oxidized by metal ions of a higher redox potential, due to the weaker binding of M‐A than M‐X. For instance, the Si in Ti_3_SiC_2_ can be easily oxidized to SiCl_4_ by CuCl_2_ (Si^4+^/Si of −1.38 V vs Cl_2_, Cu^2+^/Cu of −0.43 V vs Cl_2_), and the evaporation of SiCl_4_ leaves diffusion paths for internal etching and improves the reaction kinetics.^[^ ^12^, ^13^ ^]^ There is also a possibility that Cu^2+^ etches the Ti atoms due to the high reactivity of bare Ti atoms, especially when using a longer etching time, higher temperature, or with blocked diffusion paths. Song et al. proposed a possible reaction mechanism, as shown in **Figure** **8**a.^[^ ^82^ ^]^ The Co^2+^ or Cu^2+^ ions in molten state etch the Al atoms in Ti_2_AlN, and the reduced Co or Cu atoms aggregate to form particles that can be later removed. At the same time, some Ti atoms are also be etched to leave vacancies. The reduced Co or Cu atoms can be anchored (Co@Ti_2‐_ *_x_* N or Cu@Ti_2‐_ *_x_* N), similar to the well‐known defects engineering to synthesize single‐atom catalysts. The whole reaction can be expressed as follows:

(1) $$\begin{array}{c} Ti_{2}AlN+CuCl_{2}\to Cu@Ti_{2-x}N+AlCl_{3}↑+TiCl_{4}+Cu\left(particles\right) \end{array}$$

Other transition metal cations can achieve a similar effect,^[^ ^81^, ^94^ ^]^ which shows that the molten salt etching route can also be an effective and straightforward way to synthesis single‐atom metal catalysts on MXenes.

As mentioned earlier, in‐plane pores on Ti_3_C_2_T*_x_* flake are prepared with molten NaCl/ZnCl_2_ salt mixtures as etchants during the synthesis. A pore preserving mechanism was proposed by Zhang et al. to explain the pore formation.^[^ ^83^ ^]^ Different from the mechanism that vacancies caused by molten salt etching, they thought the defects present after etching were derived from precursor's original structural/crystal defects. The pores could be well preserved with the physical support of the larger individual crystals (e.g., NaCl) during cooling. Otherwise, they would collapse and disappear after cooling if only ZnCl_2_ is used.

The reasons for the reduction of metal ions and the in‐plane pore generation in MXenes in during post‐processing have also been studied. Ti_3_C_2_T*_x_* can be oxidized to TiO_2_ in Cu^2+^ solutions as mentioned above. Ren et al. believed that the real oxidant is the dissolved O_2_ in water, while the Cu^2+^ cations act as catalysts.^[^ ^85^ ^]^ However, a charge transfer mechanism has been proposed by Xie et al., where the reduction of metal ions is accompanied by valence state changes of Ti in Ti_3_C_2_T*_x_*.^[^ ^89^ ^]^ Ti_3_C_2_T*_x_*‐based films were fabricated to remove heavy metal ions, and both negatively and positively charged ions were found to have significant removal efficiency, which rules out simple adsorption with electrostatic interactions. With the HCrO_4_^−^ removal, the interlayer spacing of Ti_3_C_2_T*_x_* is increased and the material becomes disordered, suggesting the intercalation of Cr species. It is interesting to find the coexistence of Cr(VI) and Cr(III) species, with a high percentage (up to 82%) for Cr(III) (Figure 8b). Meanwhile, an increased fraction of Ti(IV)‐O is found by fitting the XPS spectra (Figure 8c), meaning the electron loss from Ti_3_C_2_T*_x_*. It is reasonably speculated that electrons are transferred from Ti_3_C_2_T*_x_* to HCrO_4_^−^, resulting in Cr(III) generation.

The degree of this charge transfer interaction between MXenes and metal ions may differ depending on the surface terminations, M elements, and ion species. In the case of HCrO_4_^−^ and Ti_3_C_2_, enhanced adsorption of HCrO_4_^−^ on ‐OH terminated Ti_3_C_2_ is indicated, with higher binding energies (−6.32 to −6.42 eV) than the O‐terminated one (0.72 to −1.53 eV).^[^ ^89^ ^]^ Based on the Bader analysis (Figure 8d), 0.51 e^−^ is calculated to transfer from ‐O terminated Ti_3_C_2_ to HCrO_4_^−^, and 1.48 e^−^ from the ‐OH terminated one. For Ag^+^ ions, stronger adsorption is found on ‐OH terminated Ti_3_C_2_ MXene (−2.26 eV), with an electron transfer of 0.7 e^−^. In the case of U(VI) removal by Ti_2_CT*_x_*, a similar charge transfer mechanism is dominant throughout the process and found to be pH‐dependent.^[^ ^95^ ^]^ U(VI) is reduced to U(IV) with the simultaneous formation of TiO_2_ over a wide pH range from 3.0 to 8.0. Under acidic conditions, the reduced U(IV) species are mononuclear complexes anchored on = TiO sites, as shown in Figure 8e. In near‐neutral conditions, the species are identified to be UO_2+_ *_x_* nanoparticles. Nevertheless, Ti_3_C_2_T*_x_* shows weaker reducibility of U(VI) than Ti_2_CT*_x_*, and a stronger electrostatic repulsion is thought to be the main reason.^[^ ^96^, ^97^, ^98^ ^]^

## 3 Metal Ions in MXene Applications

### 3.1 Applications in Supercapacitors

MXenes are promising electrode materials in supercapacitors, with superhigh volumetric and gravimetric capacitance (up to ≈1500 F cm^−3^ and ≈400 F g^−1^, respectively).^[^ ^99^, ^100^ ^]^ Due to their superior flexibility, optical properties, and electronic conductivity, they are suitable for fabrication into various supercapacitor devices, such as micro‐supercapacitors^[^ ^101^, ^102^, ^103^ ^]^ and transparent solid‐state supercapacitors.^[^ ^104^, ^105^ ^]^ It has been demonstrated that various cations can be electrochemically reversibly intercalated into MXene and occupy redox‐active sites to participate in energy storage. For MXenes, ions are normally adsorbed on the edges or the outer surface of the particle firstly, and then intercalated into layers. The intercalated ions are always accompanied by a hydrated shell in aqueous electrolytes, and this causes an increase in the interlayer spacing of MXene, while an increased electrostatic attraction between the Ti_3_C_2_T*_x_* sheets and intercalated cations during discharging leads to a contraction instead. The response of electrodes, which is determined by the competition between contraction and expansion, differs with different metal ions.^[^ ^106^, ^107^ ^]^ For electrode materials, zero deformation is preferred to maintain the electrode structure and achieve a prolonged lifetime and stable cycling.

MXenes store charges by ion adsorption and intercalation, and the charge storage capacitance is dominated by the specific charge storage mechanism. MXenes are regarded as intrinsically pseudo‐capacitive materials, due to their surface chemistry and layered structure. However, the charge storage mechanism can be related to EDL (i.e., electrostatic interactions) or pseudo‐capacitive one (i.e., surface redox reaction or intercalation), or a combination of both, which depends on electrolyte components. In acidic electrolytes (e.g., H_2_SO_4_), a pseudo‐capacitive mechanism is recognized. Measurements for Ti_3_C_2_T*_x_* in H_2_SO_4_ have shown that the reduced oxidation state of Ti changes with the protonation of oxygen functional groups:^[^ ^109^, ^110^ ^]^

(2) $$\begin{array}{c} Ti_{3}C_{2}O_{x}{\left(OH\right)}_{y}F_{z}+1/2xe^{-}+1/2xH^{+}\;\to \;Ti_{3}C_{2}O_{1/2x}{\left(OH\right)}_{y+1/2x}F_{z} \end{array}$$

**Figure 9:**
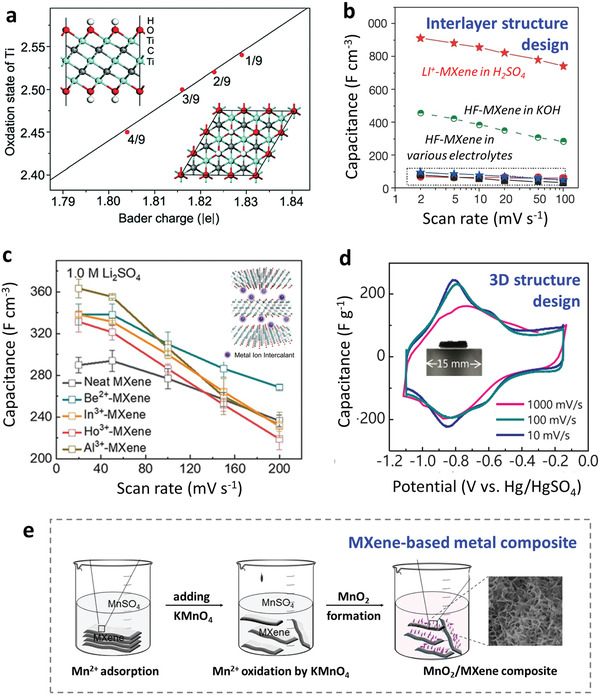
Applications in supercapacitors. a) Bader charge on Ti oxidation state changes with different H coverages. Insets show the side and top views of partially protonated Ti_3_C_2_O_2_. Reproduced with permission.^[^ ^107^ ^]^ Copyright 2020, Royal Society of Chemistry. b) Comparison of the rate performance for LiF/HCl‐produced MXene and HF‐produced MXene. Reproduced with permission.^[^ ^37^ ^]^ Copyright 2014, Springer Nature. c) The volumetric‐specific capacitance of pure Ti_3_C_2_T*_x_* MXene, Be^2+^‐MXene, Al^3+^‐MXene, In^3+^‐MXene, Ho^3+^‐MXene electrodes in 1.0 Ｍ Li_2_SO_4_ electrolyte at scan rates ranging from 20 to 200 mV s^−1^. Reproduced with permission.^[^ ^42^ ^]^ Copyright 2020, Wiley‐VCH. d) Cyclic voltammetry profiles of MXene powder and hydrogel at different scan rates. Reproduced with permission.^[^ ^28^ ^]^ Copyright 2019, Wiley‐VCH. e) Schematic of MnO_2_/MXene composite synthesis using the interaction between Mn ions and MXene. Reproduced with permission.^[^ ^108^ ^]^ Copyright 2019, American Chemical Society.

The DFT calculations in **Figure** **9**a indicate a linear relation between the Ti oxidation state change and the atomic Bader charge at different O/OH ratios and electrode potential.^[^ ^111^ ^]^ The number of active sites determines the pseudo‐capacitance in H_2_SO_4_, and an ultra‐high capacitance of 1190 F g^−1^ is predicted for Ti_3_C_2_O*_x_*. At low reaction rate, there are minor differences between the ion adsorption and intercalation in H_2_SO_4_. In neutral electrolytes (e.g., Li_2_SO_4_, K_2_SO_4_, MgSO_4_), the EDL charge storage is the main contribution, no matter for ion adsorption and intercalation. The different mechanisms for H_2_SO_4_ and for neutral electrolytes are induced by the strong interactions between water solvent molecules and cations. The hydrated shell is thought to hinder the orbital coupling of the intercalated or adsorbed cations with the MXenes.^[^ ^66^ ^]^ For this reason, the pseudo‐capacitive reaction cannot occur within the applicable potential window, resulting in solely EDL formation, even under confinement of MXene layers. Recently, the electrochemical behavior of Ti_3_C_2_T*_x_* MXene in water‐in‐salt electrolytes (e.g., 19.8 m LiCl/H_2_O) is reported. Separated sharp redox peaks in CV curves and an abrupt change of the interlayer spacing (11.0–12.9 Å) are observed. The diffusion‐controlled (de‐)intercalation of solvated cations causes these changes. This behavior is distinct from typical EDL or pseudocapacitive charge storage. Overall, more efforts are required for a fundamental understanding of charge storage in MXenes.

Pure MXene electrodes prepared by HF etching have an unsatisfactory performance in various electrolytes, including H_2_SO_4_ (Figure 9b),^[^ ^22^ ^]^ but by taking advantage of their interactions between metal ions, their potential can be realized. Cation intercalation during etching has proven to produce a significant improvement. The Ti_3_C_2_T*_x_* synthesized in LiF/HCl has outstanding performance in a H_2_SO_4_ solution, with a high capacitance of 900 F cm^−3^ at 2 mV s^−1^ and good rate handling ability (Figure 9b).^[^ ^37^ ^]^ As mentioned earlier, Li^+^ intercalation improves the exfoliation kinetics. The obtained MXenes has a higher conductivity, larger interlayer spacing, and higher content of ‐O terminations, which together improve the intercalation and surface redox reaction pseudo‐capacitance. Additionally, it is a common strategy to change the interlayer structure by ion pre‐intercalation to expose more active sites and facilitate ion diffusion.^[^ ^43^ ^]^ For example, MXene electrodes with controlled interlayer spacings are prepared by pre‐intercalating various metal cations to match the size of the charge carriers in neutral electrolytes (Figure 9c).^[^ ^42^ ^]^ As a result, Al^3+^‐MXene and Be^2+^‐MXene films have improved volumetric capacitances (*C*_v_) of 351.09 F cm^−3^, and 330.7 F cm^−3^ at 20 mV s^−1^ in 1.0 m Li_2_SO_4_, respectively. Compared with neat MXene, the Be^2+^‐MXene also has C_v_ increases of 16.4% in Na_2_SO_4_, 19.2% in K_2_SO_4,_ and 30.1% in ZnSO_4_. Higher capacitance retention of 81.8% in K_2_SO_4_ is presented at 200 mV s^−1^. The surface chemistry can also be changed to improve the capacitance. Typically, more oxygen‐containing functional groups can be introduced on the MXenes surface by immersing them in metal hydroxide solutions.^[^ ^112^ ^]^ A high capacitance of 500 F g^−1^ at 1 mV s^−1^ was reported after K^+^ ion intercalation and calcination, where only ‐O functional groups terminate the surface.^[^ ^53^ ^]^

A 3D MXene electrode can be fabricated to address the restacking problem of 2D nanosheets and shorten the mass transfer path by using the crosslinking of metal ions.^[^ ^29^ ^]^ A Ti_3_C_2_T*_x_* MXene hydrogel electrode prepared by Fe^2+^ crosslinking can achieve an excellent rate performance (≈226 F g^−1^), and its typical pseudocapacitive behavior is well maintained at 1 V s^−1^, as shown in Figure 9d.^[^ ^28^ ^]^ Ding et al. used a Mg^2+^‐MXene aerogel as electrodes and PVA/H_2_SO_4_ as a gel electrolyte to construct quasi‐solid state micro‐supercapacitors.^[^ ^68^ ^]^ As a result, a high areal‐specific capacitance (409.3 mF cm^−2^ at 5 mV s^−1^) was obtained, as well as high areal energy densities (7.4–21.6 μWh cm^−2^) and high areal power densities (0.1–1.1 µW cm^−2^). In addition, MXenes are regarded as ideal 2D supports to couple with other pseudocapacitive materials, and their interactions with metal ions help the fabrication of composite electrodes.^[^ ^113^, ^114^ ^]^ As illustrated in Figure 9e, a MnO_2_/Ti_3_C_2_T*_x_* composite is synthesized by impregnating Ti_3_C_2_T*_x_* MXene in a MnSO_4_ solution, with KMnO_4_ being reduced.^[^ ^108^ ^]^ After annealing, the composite electrode had a capacitance of 212.1 F g^−1^ at 1 A g^−1^. Similarly, other composite electrodes have been extensively investigated, such as Ti_3_C_2_/MoS_2_,^[^ ^115^ ^]^ Ti_3_C_2_/CuS,^[^ ^116^ ^]^ Ti_3_C_2_/FeOOH quantum dots,^[^ ^117^ ^]^ Ti_3_C_2_/NiCoFe‐LDH,^[^ ^118^ ^]^ Ti_3_C_2_/NiFe‐LDH,^[^ ^119^, ^120^ ^]^ Ti_3_C_2_/Ni_3_S_2_,^[^ ^121^ ^]^ Ti_3_C_2_/Fe_3_O_4_,^[^ ^122^ ^]^ Ti_3_C_2_/Ni_2_CO_3_(OH)_2_,^[^ ^123^ ^]^ etc.

### 3.2 Applications in Metal‐Ion Batteries

Rechargeable lithium‐ion batteries (LIBs) are one of the most significant energy storage devices, and are widely used in portable electronic devices and the new electric vehicles. The pursuit of higher energy densities and the limited lithium resources have promoted the exploration of new electrode materials and non‐lithium‐ion batteries, including sodium‐ion batteries (SIBs), potassium‐ion batteries (PIBs), calcium‐ion batteries (CIBs), etc.^[^ ^61^ ^]^ MXenes are new materials that can accommodate the insertion of various metal ions between their layers. As electrode materials, they are predicted to provide remarkable theoretical capacities (e.g., 447.8 mAh g^−1^ for LIBs, 351.8 mAh g^−1^ for SIBs, 191.8 mAh g^−1^ for PIBs, 319.8 mAh g^−1^ for CIBs, based on bare Ti_3_C_2_).^[^ ^124^, ^125^, ^126^, ^127^, ^128^, ^129^, ^130^ ^]^ Given that the metal ions play a crucial role as charge carriers in metal‐ion batteries (MIBs), their interactions with MXenes, especially in electrochemistry, become extremely important.

**Figure 10:**
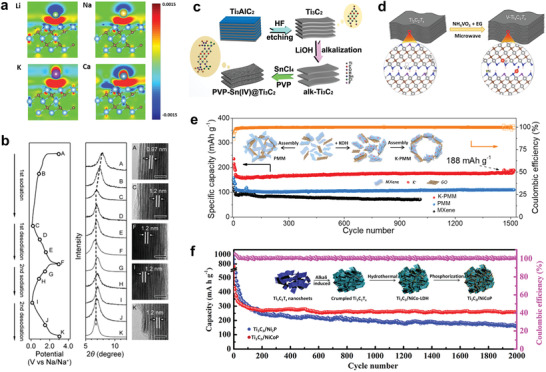
Applications in metal‐ion batteries, with interlayer structure design, 3D structure design, and MXene composite strategy. a) Bonding charge density for Li, Na, K, and Ca ions in the Ti_3_C_2_T*_x_* system. These bonding charge distributions clearly show charge transfer from the atoms to the Ti_3_C_2_ monolayer. Reproduced with permission.^[^ ^130^ ^]^ Copyright 2014, American Chemical Society. b) XRD patterns and TEM images of Ti_3_C_2_T*_x_* upon sodiation and de‐sodiation. The scale bars in the TEM images indicates 5 nm. Reproduced with permission.^[^ ^67^ ^]^ Copyright 2016, American Chemical Society. c) Schematic of the fabrication process of (polyvinylpyrrolidone, PVP)‐Sn(IV)@Ti_3_C_2_. With the PVP surfactant, the adsorbed Sn(IV) can be uniformly distributed in the alk‐Ti_3_C_2_ matrix. Reproduced with permission.^[^ ^131^ ^]^ Copyright 2016, American Chemical Society. d) Illustration of the microwave‐assisted approach for the N (V)‐modified Ti_3_C_2_T*_x_*. V_Ti_ represents a Ti vacancy. Reproduced with permission.^[^ ^132^ ^]^ Copyright 2020, American Chemical Society. e) Cycling performance at 100 mA g^−1^ for K‐PMM, PMM, and MXene films. The inset Figure is a diagram of the preparation process of K‐PMM and PMM. Reproduced with permission.^[^ ^133^ ^]^ Copyright 2021, Elsevier. f) Cycling performance of Ti_3_C_2_, Ti_3_C_2_/Ni_2_P, and Ti_3_C_2_/NiCoP electrodes. The inset Figure in (f) is an illustration of the synthesis of the Ti_3_C_2_/NiCoP composite. Reproduced with permission.^[^ ^134^ ^]^ Copyright 2019, Royal Society of Chemistry.

Given the preferred ion adsorption on the surface, M_2_X with a higher effective surface area is expected to provide higher gravimetric capacities than M_3_X_2_ and M_4_X_3_.^[^ ^129^ ^]^ That is to say, compared with Ti_3_C_2_ and Ti_4_C_3_, Ti_2_C has a higher theoretical gravimetric capacity.^[^ ^61^ ^]^ In addition, ‐OH and ‐F terminations are believed to block ion diffusion and lower the theoretical capacities, while ‐O functional groups provide additional capacity.^[^ ^25^, ^135^ ^]^ For example, once terminated with ‐F or ‐OH, the theoretical capacity is reduced to 130 or 67 mAh g^−1^ for Li^+^ storage.^[^ ^136^ ^]^ The different theoretical specific capacities shown for different metal ions can be understood by referring to the maximum adatom content and the corresponding charge transfer, which determines the electronic properties after ion adsorption.^[^ ^130^ ^]^ **Figure** **10**a illustrates the bonding charge density for adatoms on bare Ti_3_C_2_ and the charge transfer from adatoms to Ti_3_C_2_. It is calculated that the amount of charge transfer are 0.21 (Li), 0.40 (Na), 0.47 (K), and 1.31 e^−^ (Ca), with the respective maximum adatom contents being 2.8, 2.2, 1.2, and 1. Moreover, the corresponding diffusion barriers are estimated to be 0.068 (Li), 0.096 (Na), 0.103 (K), and 0.118 (Ca) eV, which are much lower than that on graphite (0.3 eV for Li). These calculations indicate that MXenes are promising electrode materials with a high rate.

Experimentally, when used the anode materials in LIBs, multilayer Ti_2_C powders prepared by 10 wt% HF gives capacities of 225 mAh g^−1^ at C/25, and 110 mAh g^−1^ at 1 C.^[^ ^62^ ^]^ To achieve satisfied performance with rational material designs, the investigation of ion storage mechanism is urgently needed. MXenes store ions by adsorption and intercalation, and generally show distorted rectangular CVs with small pairs of redox peaks. In organic electrolytes, partially or complete de‐solvation of cations occurs at the MXene‐electrolyte interface or in intercalation, due to the lower solvation energy than hydration. The solvents would decompose and solid electrolyte interface (SEI) forms under the wide potential window. In most cases, those cations adsorbed on the MXene surface have solvation shells, and the complete charge separation results in EDL storage. However, those intercalated cations are usually partially or fully de‐solvated, and a donor band forms due to the orbital overlap with a close contact of MXene‐cation. This orbital hybridization allows charge transfer between MXenes and intercalated cations, and enhances the capacity. In 1.0 m LiPF_6_‐ethylene carbonate/dimethyl carbonate (LiPF_6_‐EC/DMC), a reversible interlayer spacing change (9.4–9.8 Å) is found, which is caused by the partially de‐solvated Li^+^ (de‐)intercalation.^[^ ^62^ ^]^ At the same time, a reversible reduction/oxidation of Ti of Ti_2_CT*_x_* is caused. As mentioned in section 2.1.3, the PC solvent enables a fully de‐solvated ion intercalation, and the increased intercalated ion number (0.93 Li^+^ per Ti_3_C_2_T*_x_*) leads to a higher capacity. In 1 m NaPF_6_‐ethylene carbonate/diethyl carbonate (NaPF_6_‐EC/DEC), the layered Ti_3_C_2_T*_x_* has a capacity of 270 mAh g^−1^ at 20 mA g^−1^ for the first Na^+^ intercalation/adsorption and a good cycle stability (100 mAh g^−1^ over 100 cycles) the first few cycles.^[^ ^67^ ^]^ Electrolyte decomposition is the main reason for the irreversible capacity in these cycles. A combination of XRD and TEM, shows that the interlayer spacing increases from 0.97 to 1.20 nm during the first sodiation, and remains unchanged in the next de‐sodiation and the second sodiation (Figure 10b). This Na^+^ pillaring effect helps subsequent (de‐)intercalation of de‐solvated Na^+^ and result in a stable cycling.

According to the above discussion, the interlayer structure can determine the intercalation kinetics of metal ions and make full use of the active sites.^[^ ^137^, ^138^, ^139^ ^]^ Luo et al. used the ion‐exchange behavior and electrostatic interactions between MXenes and metal ions to produce Ti_3_C_2_ decorated with Sn^4+^ ions.^[^ ^140^ ^]^ As shown in Figure 10c, the initial alkalization of MXene promotes Li^+^ intercalation and Sn^4+^ intercalation is achieved by ion exchange, where a surfactant (polyvinylpyrrolidone, PVP) helps produce a uniform distribution of Sn^4+^. As a result, an outstanding electrochemical performance was achieved, with an ultrahigh capacity (1375 mAh cm^−3^, 635 mAh g^−1^ at 100 mA g^−1^) and excellent cycling stability with a 94.3% capacity retention after 200 cycles (500 mA g^−1^). Here, the Sn (Li*_x_* Sn) and Ti_3_C_2_ (Ti_3_C_2_T*_x_* Li*_x_*) both contribute to the capacity. Similarly, Wang et al. produced Sn‐intercalated V_2_C MXene (c‐LP: 19 Å) which had a stable discharge capacity of 690.8 mAh g^−1^ at 1 A g^−1^, and 205.2 mAh g^−1^ at 20 A g^−1^, when used as the anode in LIBs.^[^ ^140^ ^]^

Doping is also powerful in changing the band structure and improving the electrochemical performance.^[^ ^132^, ^141^, ^142^ ^]^ N (V)‐modified Ti_3_C_2_T*_x_* MXene was prepared by microwave irradiation Ti_3_C_2_T*_x_* powders in NH_4_VO_3_/EG (ethylene glycol), as shown in Figure 10d.^[^ ^132^ ^]^ The V atoms were doped into Ti_3_C_2_ lattice to occupy Ti vacancies, and bind with ‐O surface groups to increase the interlayer spacing, through the interaction of VO^2+^ ions with Ti_3_C_2_T*_x_*. Meanwhile, the surface was modified by N, through the interaction with NH_4_^+^. The co‐doping of V and N increases the capacity from 180.1 to 251.3 mAh g^−1^ at 0.1 C, and improves the cycling durability. It has been reported that V_2_C MXene with a controlled interlayer spacing exhibits the Li‐ion capacity to 686.7 mAh g^−1^ at 0.1 A g^−1^. The performance is found to be improved to 1117.3 mAh g^−1^, by intercalation with Co ions.^[^ ^141^ ^]^ Here, strong interaction between the VO^2+^ ions and MXene is confirmed, as evidenced by the existence of V‐O‐Co bonding.

3D structure design is also frequently used to improve the kinetics and shorten the mass transfer length, thus improving the rate performance, especially in SIBs.^[^ ^143^, ^144^ ^]^ As mentioned earlier, alkali metal hydroxides produce a crumpled 3D structure of MXenes, and by using this property, Na^+^ ion storage can reach a specific capacity of 267 mAh g^−1^ (25 mA g^−1^) in the 1^st^ cycle and 170 mAh g^−1^ in the 2^nd^ cycle after being treated with NaOH. One treated with LiOH still display had a capacity of ≈160 mAh g^−1^ (0.1 A g^−1^), after 300 cycles. The 3D assembly of Ti_3_C_2_T*_x_* MXene can be achieved with the assistance of GO sheets and ethylenediamine (EDA),^[^ ^145^ ^]^ and its microstructure could be regulated by the interaction of metal ions with MXene sheets. Zhao et al. designed a 3D Ti_3_C_2_T*_x_* network with a local laminated structure, by adding KOH in the assembly process.^[^ ^133^ ^]^ It showed a excellent long‐term cycling as the anode of SIB, with a capacity retention of 188 mAh g^−1^ after 1500 cycles (100 mA g^−1^), as shown in Figure 10e.

Similar to their use in supercapacitors, the use of MXene hybrid materials is another common way to provide a highly conductive framework for high‐capacity active materials.^[^ ^146^ ^]^ For example, through the adsorption of Sn^2+^ on Ti_3_C_2_ sheets, SnO*_x_* @Ti_3_C_2_ anode was prepared by a hydrothermal reaction, which had a much higher Li‐storage capacity of ≈450 mAh g^−1^ than SnO (≈63 mAh g^−1^) and Ti_3_C_2_ anodes.^[^ ^139^ ^]^ As illustrated in Figure 10f, NiCo‐LDH materials were loaded on crumpled MXene, by introducing Ni^2+^, Co^2+^, and urea to an alkali‐Ti_3_C_2_ suspension, followed by hydrothermal treatment.^[^ ^134^ ^]^ Then Ti_3_C_2_/NiCoP composite was then prepared by phosphorization with NaH_2_PO_2_. When used as the anode material in SIBs, it retained a specific capacity of 261.7 mAh g^−1^ at 1 A g^−1^ after 2000 cycles. Zou et al. used the self‐reduction of Ag^+^ ions on MXene form a Ti_3_C_2_‐Ag hybrid, which had an extraordinary long cycle lifetime (>5000 cycles) with a high Li‐storage capacity of 310 mAh g^−1^ at 1 C, as well as a good rate performance (150 mAh g^−1^ at 50 C).^[^ ^147^ ^]^ Other hybrid electrodes have been fabricated for MIBs, such as Ti_3_C_2_/Fe_2_O_3_,^[^ ^148^, ^149^, ^150^, ^151^ ^]^ Ti_3_C_2_/W‐doped Nb_2_O_5_,^[^ ^152^ ^]^ Ti_3_C_2_/MoO_3‐_ *_x_*,^[^ ^153^ ^]^ and Ti_3_C_2_/GeO*_x_* ^[^ ^154^ ^]^ for LIBs, and Ti_3_C_2_/FeS_2_,^[^ ^155^ ^]^ Ti_3_C_2_/Sb_2_O_3_,^[^ ^156^ ^]^ Ti_3_C_2_/CoNiO_2_,^[^ ^157^ ^]^ and Ti_3_C_2_/SnS^[^ ^158^ ^]^ for SIBs, etc.

In recent years, MXene based metal ion hybrid capacitors (MICs) are attracting increasing research attention, due to the advantages of both high energy density and power density.^[^ ^159^, ^160^ ^]^ This kind of device is composed of one battery‐type electrode and the other capacitor‐type electrode. In most cases, the battery‐type redox reaction occurs on the anode side, and capacitive reaction occurs on the cathode side.^[^ ^161^, ^162^, ^163^ ^]^ Sometimes, there is a reverse situation, and it depends on the potentials of the two reactions. As introduced before, MXenes demonstrate excellent ion storage performance, so they can be anodes in MICs. Luo et al. fabricated pre‐intercalated Ti_3_C_2_ MXene (CTAB‐Sn(IV)@Ti_3_C_2_) with cetyltrimethylammonium bromide (CTAB) and Sn^4+^ pillaring.^[^ ^164^ ^]^ When coupling the CTAB‐Sn(IV)@Ti_3_C_2_ anode and activated carbon (AC) cathode, the lithium‐ion hybrid capacitor (LIC) displayed the specific capacitance of 268 F g^−1^ at 0.2 A g^−1^ (based on the anode mass), and good capacity retention of 71.1% after 4000 cycles at 2 A g^−1^. Fan et al. prepared nitrogen‐doped porous Ti_3_C_2_T*_x_* (N‐Ti_3_C_2_T*_x_*) by using the template of melamine‐formaldehyde (MF) nanospheres.^[^ ^165^ ^]^ With the AC cathode, the constructed sodium ion hybrid capacitors (SICs) achieved a gravimetric energy and power density of 101.6 Wh kg^−1^ and 3269 W kg^−1^, respectively. Song et al. designed S‐doped Ti_3_C_2_T*_x_* aerogels (SMGA) by crosslinking S/Ti_3_C_2_T*_x_* composites and GO sheets, with linking agents (e.g., amino‐propyltriethoxysilane, Mn^2+^, Fe^2+^, Zn^2+^, and Co^2+^).^[^ ^166^ ^]^ The resulting electrodes exhibited excellent Na^+^ ion storage (1.26 mAh cm^−2^) with a high mass‑loading (12.3 mg cm^−2^). The constructed SMGA//AC SICs displayed a capacity retention of 92.9%, and maintained a capacity of 93.7 mAh g^−1^ (based on the anode mass) after 1600 cycles. Zhao et al. designed a 3D K^+^‐pre‐intercalated Ti_3_C_2_T*_x_* MXene by alkali treatments to store K^+^ ions.^[^ ^167^ ^]^ The assembled 3D K^+^‐Ti_3_C_2_T*_x_*//AC potassium ion hybrid capacitors (PICs) achieved a high energy density of 163 Wh kg^−1^ and a long‐term cycling (up to 10 000 cycles), with the formation of a thin and inorganic‐rich SEI in 4 m KFSI electrolytes. Owing to their fast redox kinetics, MXenes can also be used as cathodes of MICs.^[^ ^168^, ^169^, ^170^, ^171^ ^]^ For instance, Li et al. designed Sn^4+^ pre‐intercalated Ti_2_CT*_x_* MXene (Sn^4+^‐Ti_2_CT*_x_*) cathodes with enlarged interlayer spacing for aqueous zinc ion hybrid capacitors (ZICs), with zinc metal anodes.^[^ ^171^ ^]^ To further shorten the diffusion length, the Sn^4+^‐Ti_2_CT*_x_* was aligned on the carbon spheres. As expected, a specific capacity of 138 mAh g^−1^ at 0.1 A g^−1^ and ultralong cycling of 12 500 cycles have been achieved.

### 3.3 Applications in Catalysis

Recently, MXenes have attracted great attention in catalysis due to their high surface area, good electronic conductivity, and metal atom cores.^[^ ^172^, ^173^ ^]^ Producing a large number of active catalysis sites on MXene is a widely adopted strategy to increase its performance as a catalyst, where its interactions with different metal ions are made full use of.^[^ ^125^, ^174^ ^]^

**Figure 11:**
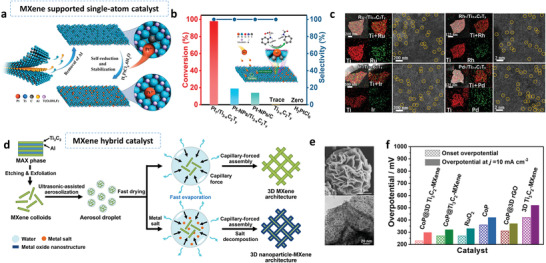
Applications of MXenes in catalysis. a) Illustration of the simultaneous self‐reduction stabilization process for preparing of Pt_1_/Ti_3‐_ *_x_* C_2_T_y_. b) Catalytic performance of the N‐formylation of aniline using different catalysts. c) HAADF‐STEM images and corresponding elemental maps of M_1_/Ti_3‐_ *_x_* C_2_T_y_ (M: Ru, Ir, Rh, and Pd). Reproduced with permission.^[^ ^179^ ^]^ Copyright 2019, American Chemical Society. d) Capillary‐forced assembly of MXene into a 3D structure and related composite systems by spray drying the aerosol droplets of colloids containing MXene. e) SEM and TEM images of the CoP@3D Ti_3_C_2_‐MXene structure. f) A comparison of the onset overpotential and overpotential of different catalysts at applied current density (*j* = 10 mA cm^−2^). Reproduced with permission.^[^ ^184^ ^]^ Copyright 2018, American Chemical Society.

First, MXene‐supported single metal atom catalysts (SACs@MXene) can be synthesized by anchoring metal atoms in cation vacancies or surface groups.^[^ ^30^, ^91^, ^175^, ^176^, ^177^, ^178^ ^]^ Zhao et al. synthesized a Pt_1_/Ti_3‐_ *_x_* C_2_T_y_ single‐atom catalyst by mixing a [PtCl_6_]^2−^ solution with a MXene (Ti_3‐_ *_x_* C_2_T_y_) suspension, and no additional reduction operation was needed (**Figure** **11**a).^[^ ^179^ ^]^ During the process, the [PtCl_6_]^2−^ ions were adsorbed on the MXene surface after mixing, and then the metal ions were self‐reduced and immobilized on the Ti vacancies. By controlling the amount of LiF used in Ti_3_C_2_T*_x_* MXene synthesis, the concentration of Ti vacancies could be regulated, as reported earlier. The valence state of the Pt atom was found to be between 0 and +2, with a higher oxidation state of Ti atoms. The Pt_1_/Ti_3‐_ *_x_* C_2_T_y_ had an outstanding catalytic performance for CO_2_ functionalization, and a nearly 100% conversion rate of the N‐formylation of aniline (Figure 11b) was achieved. This method is also suitable for preparing other MXene‐supported noble‐metal SACs, such as Ru_1_/Ti_3‐_ *_x_* C_2_T_y_, Ir_1_/Ti_3‐_ *_x_* C_2_T_y_, Rh_1_/Ti_3‐_ *_x_* C_2_T_y_, and Pd_1_/Ti_3‐_ *_x_* C_2_T_y_, (Figure 11c) as well as non‐noble‐metal SACs, such as Fe_1_/Ti_3‐_ *_x_* C_2_T_y_, Co_1_/Ti_3‐_ *_x_* C_2_T_y_, Ni_1_/Ti_3‐_ *_x_* C_2_T_y_. SACs on MXene can also be prepared directly from MAX phases by molten salt etching, as mentioned earlier. The synthesized Cu SACs@MXene has a high faradaic efficiency (59.1%) in producing CH_3_OH in electrocatalytic CO_2_ reduction (CO_2_RR).^[^ ^180^ ^]^ Ramalingam et al. prepared Ru SACs@MXene by thermal annealing, after mixing a Ti_3_C_2_T*_x_*, Ru^3+^ solution with thiourea.^[^ ^181^ ^]^ The Ru^3+^ ions interact with the ‐O/‐OH terminations on the MXene surface, and are then reduced by an annealing treatment. For the hydrogen evolution reaction (HER), a low overpotential (76 mV) was achieved at a current density of 10 mA cm^−2^.

Second, a wide range of MXene hybrids can be synthesized by the reaction of metal salts on the MXene matrix.^[^ ^124^, ^182^, ^183^, ^184^, ^185^, ^186^ ^]^ As shown in Figure 11d, Co_3_O_4_@MXene, SnO_2_@MXene, MnTiO_3_@MXene, Pt@MXene, and Ag@MXene catalysts were prepared by the aerosol spray drying of MXene colloids dissolved in the corresponding metal salts, such as Co(OAC)_2_·4H_2_O, SnCl_4_, Mn(CH_3_COO)_2_·4H_2_O, H_2_PtCl_6_·6H_2_O, or AgNO_3_.^[^ ^184^ ^]^ The metal ions are distributed uniformly on the 3D MXene matrix with strong interactions, and the salt decomposes into the corresponding metal oxide or metal. Among them, Co_3_O_4_@MXene can be converted into CoP@MXene (Figure 11e) by phosphorization at high temperatures. This hybrid catalyst has a high electrocatalytic activity in the oxygen evolution reaction (OER) and the HER, with much lower overpotentials of 280 mV (10 mA cm^−2^) for OER and 128 mV for HER in 1 m KOH (Figure 11f). Yu et al. fabricated FeNi‐LDH/Ti_3_C_2_‐MXene with the co‐precipitation of Fe^3+^ and Ni^2+^ in a MXene dispersion,^[^ ^185^ ^]^ during which the interaction between ions and negatively charged MXene facilitated the nucleation and anchoring of FeNi‐LDH nanostructures on the MXene surface.

Recently, ordered surface‐supported intermetallic compounds (IMCs, e.g., Pt_3_Ti and Pt_3_Nb) have been prepared using the reactive metal‐support interactions on Pt/MXene catalysts. For instance, Ti_3_C_2_T*_x_* was first impregnated with a Pt(NH_3_)_4_(NO_3_)_2_ solution to form a fresh Pt/Ti_3_C_2_ catalyst and then reduced at 550 °C in 5% H_2_/N_2_ for at least 0.5 h. In this process, Pt atoms are not simply embedded in the Ti_3_C_2_T*_x_* lattice, but form a bimetallic alloy with surface Ti atoms, which exhibits a superior HER performance in acidic media to give a high ^*^H adsorption strength.^[^ ^187^, ^188^ ^]^ Pt_3_Nb supported on Nb_2_CT*_x_* was prepared in a similar way, and has been reported to provide a higher H_2_O activation than the original catalyst or pure Nb_2_CT*_x_*.^[^ ^177^ ^]^ However, Pt atomically thin nanolayers (ATNLs) rather than Pt‐Mo alloys are formed on the Mo_2_TiC_2_T*_x_* surface, which is explained by the preferential formation of interfacial Mo‐Pt bonds and the lower interface formation energy of a Pt nanolayer than a nanoparticle.^[^ ^189^ ^]^ The Pt ATNLs on Mo_2_TiC_2_T*_x_* show a stable conversion (7%) and a high selectivity (>98%) for C_2_ products in the non‐oxidative methane coupling reaction.

### 3.4 Applications in Water Treatment

Water is indispensable for life. However, the rapid development of the global economy has caused massive water pollution and waste. Investigations suggest that two million tons of sewage and other wastewater are discharged globally every day. Many water pollutants (e.g., heavy metal ions, organic chemicals, nutrient substances) have long‐term negative effects on water quality and threaten human health. Therefore, water/wastewater treatment is of great importance to maintain water resources.^[^ ^190^ ^]^ The non‐toxic MXene have received growing attention due to their high specific surface area and abundant surface groups, which give them them and their derives excellent performance as adsorbents and water purification membranes.^[^ ^191^, ^192^, ^193^, ^194^, ^195^, ^196^, ^197^ ^]^

**Figure 12:**
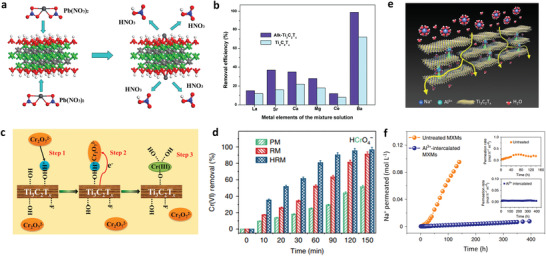
Applications of MXenes in water treatment, especially heavy metal ion removal and water purification membranes. a) Schematic of Pb^2+^ adsorption to form Ti_3_C_2_(O_2_H_2‐2m_Pb_m_). Reproduced with permission.^[^ ^198^ ^]^ Copyright 2015, American Chemical Society. b) The removal efficiency of solute elements in simulated nuclear wastewater due to adsorption by Alk‐Ti_3_C_2_T*_x_* and Ti_3_C_2_T*_x_*. Reproduced with permission.^[^ ^199^ ^]^ Copyright 2018, Royal Society of Chemistry. c) Illustration of the removal mechanism of Cr(VI) from water by Ti_3_C_2_T*_x_* sheets. Reproduced with permission.^[^ ^200^ ^]^ Copyright 2015, American Chemical Society. d) Time‐online profiles for removal of HCrO_4_^−^ from water. Error bars represent systematic errors in the measurements. Reproduced with permission.^[^ ^89^ ^]^ Copyright 2019, Springer Nature. e) Schematic of Al^3+^ intercalated MXene membranes (MXMs) for effective ion sieving. The strong interaction between Al^3+^ ions and MXene layers determines the d‐spacing. Hydrated cations, such as Na^+^, are rejected, while the water molecules permeate the membrane. f) Time‐dependent Na^+^ permeation through untreated MXMs and Al^3+^‐intercalated MXMs, indicating good long‐term stability of the Al^3+^‐intercalated MXMs with a much lower ion permeation rate. Na^+^ permeation rates (inset) through untreated MXMs and Al^3+^‐intercalated MXMs as a function of time. Reproduced with permission.^[^ ^49^ ^]^ Copyright 2020, Springer Nature.

As adsorbents, MXenes have an excellent removal efficiency and selectivity for heavy metal ions (HMIs) with the strong absorption and reducing ability.^[^ ^201^ ^]^ Generally, the concentration of toxic HMIs (e.g., Pb^2+^,^[^ ^202^, ^203^, ^204^, ^205^ ^]^ Hg^2+^,^[^ ^206^ ^]^ Ba^2+[^ ^199^, ^207^ ^]^) in water is far lower than that of light metal ions (Li^+^, Na^+^, K^+^, Mg^2+^, Ca^2+^). This means that the interactions of HMIs with MXenes are strong enough to break the shielding effect of water molecules or coordinating anions, with the formation of strong bonds. As illustrated in **Figure** **12**a, strong chemical bonds are formed between Pb atoms and O atoms on Ti_3_C_2_T*_x_* due to the formation energy of Ti_3_C_2_(O_2_H_2‐2m_Pb_m_) being lower than that of Pb(NO_3_)_2_.^[^ ^198^ ^]^ Moreover, alkali‐MXene has a better efficiency for replacing F groups and metal ion (Li^+^, Na^+^, K^+^) intercalation. Experimentally, a high Pb(II) ion uptake capacity (140 mg g^−1^) is achieved, with a high selectivity when other cations (e.g., Ca(II) and Mg(II)) are also present.^[^ ^208^ ^]^ Similarly, alkali MXene has a Ba(II) adsorption capacity of 46.46 mg g^−1^, three times that of the non‐modified one.^[^ ^199^ ^]^ It also has a ≈99% removal efficiency in a complex solution containing other elements (La, Ce, Ca, Mg, Ce) contained (Figure 12b). Based on the reduction or charge transfer mechanism, some HIMs (e.g., Cu(II),^[^ ^86^ ^]^ Cd(II), Cr(VI),^[^ ^200^ ^]^ U(VI),^[^ ^88^ ^]^) and some oxidants (e.g., K_3_[Fe(CN)_6_], KMnO_4_, AuCl_4_^−^)^[^ ^200^ ^]^ can also be removed. The residual concentration of Cr(VI) can be reduced to 5 ppb after removal by Ti_3_C_2_T*_x_* sheets, which meets the standard for drinking water. The removal capacity is as high as 250 mg g^−1^ and is pH‐dependent. In low pH or neutral solutions, Cr(VI) is reduced to Cr(III), which is anchored on the MXene surface (Figure 12c). Meanwhile, the valence state of Ti in MXene increases. The removal of Cr(VI) as cationic HCrO_4_^−^ is completed in 2 h (Figure 12d) by a RGO‐intercalated TI_3_C_2_T*_x_* film.^[^ ^89^ ^]^

As water purification membranes, Ti_3_C_2_T*_x_* laminates show excellent water flux and good ion sieving activity.^[^ ^209^ ^]^ This was first reported by Ren et al., who showed that a Ti_3_C_2_T*_x_* film had a water flux of 37.4 L Bar^−1^ h^−1^ m^−2^ with different ion permeation rates for Na^+^, Li^+^, K^+^, Ca^2+^, Ni^2+^, Mg^2+^ and Al^3+^.^[^ ^210^ ^]^ Given that ions permeate through the interlayer in the membranes, ions can be adsorbed on both surfaces of MXene interlayer walls, if the radius of hydrated ions are small. In this case, larger interlayer spacing can be obtained by Na^+^ ion permeation than Ca^2+^ and Al^3+^ ions, as the ions with smaller hydrated radius and lower charges are able to form double layer adsorption. Moreover, the electrostatic attraction between MXene sheets and highly charged ions may cause the interlayer contraction. As a result, the permeation rate of Na^+^ is about 25 times that of Al^3+^ and 7 times that of Ca^2+^. Pandey et al. fabricated an Ag@MXene membrane by self‐reduction of Ag^+^ ions on MXene sheets.^[^ ^211^ ^]^ Compared to pure MXene membrane, an increased water flux is achieved, with 92.32% and 79.93% rejection for methyl green and rhodamine B. Additionally, bacteria growth was inhibited by the Ag particles. To prevent swelling and improve the mechanical stability, MXene membranes have been intercalated with cations and crosslinking.^[^ ^49^, ^212^ ^]^ Wang et al. prepared an Al^3+^ intercalated‐MXene membrane (Figure 12e). This membrane had a high Na^+^ rejection rate of 96.5%, and maintained the structure integrality without swelling up to 400 h (Figure 12f).^[^ ^49^ ^]^ It is considered that the outstanding non‐swelling property is achieved by the strong interactions between Al^3+^ and MXene sheets, which contributes a fixed interlayer space.

### 3.5 Applications in Other Fields

**Figure 13:**
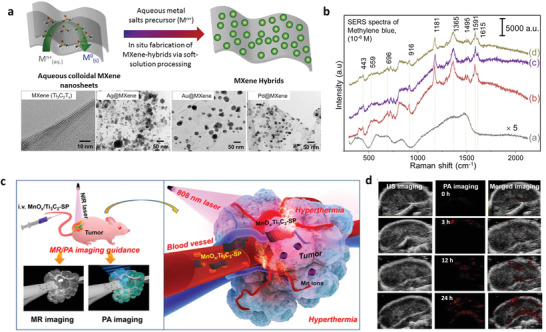
Applications of MXenes in other fields. a) Graphical representation of *in‐situ* one‐step solution processing synthesis of Ag, Au, and Pd@MXene hybrids by soft‐solution processing. The inset is TEM images of MXene, Ag@MXene, Au@MXene, and Pd@MXene. b) Raman spectra of Ti_3_C_2_T*_x_* after soaking in MB dispersed in ethanol and subsequent drying. SERS spectra of MB with (red) Ag@, (purple) Au@, and (yellow) Pd@MXene. Reproduced with permission.^[^ ^93^ ^]^ Copyright 2016, Springer Nature. c) Schematic of theranostic functions of MnO*_x_*/Ti_3_C_2_‐SP composite sheets, i.e., MR/PA imaging‐guided efficient tumor ablation of cancer. d) In vivo PA Imaging of MnO_x_/Ti_3_C_2_ nanosheets. Reproduced with permission.^[^ ^214^ ^]^ Copyright 2017, American Chemical Society.

In addition to the above applications, MXenes and MXene hybrids have been used in electromagnetic interference shielding, as sensors, and in therapeutic biomedicine. Liu et al. used the intercalation of Al^3+^ ion with additional ionic bonding to fabricate a MXene film with both flexibility and high strength.^[^ ^50^ ^]^ The tensile strength was improved from 28.7 to 83.2 MPa, while the electronic conductivity remained at 265 600 S m^−1^. As a result, a high EMI shielding performance (>80 dB) was obtained. MXene foams produced by Zn^2+^ crosslinking, as discussed earlier, had a shielding performance of 51.0 dB at a thickness of only 85 µm.^[^ ^74^ ^]^ Satheeshkumar et al. used the self‐reduction of specific metal ions on MXene, and synthesized Ag, Au, and Pd@MXene hybrids by one‐step solution processing, as illustrated in **Figure** **13**a.^[^ ^93^ ^]^ Metal nanoparticles were uniformly distributed on the surface, and a surface enhanced Raman scattering (SERS) effect was demonstrated, which can be used in sensors, catalysis, and the biomedine. For instance, methylene blue (MB) is used as a chemical indicator, dye, biological stain, and medicine. The signal of adsorbed MB on a NP@MXene surface can be unequivocally detected in Raman spectra, even when its concentration of MB is only 10^−6^ m (Figure 13b). Zhang et al. used the fluorescence quenching of MXene quantum dots (QDs) by Fe^3+^, and fabricated the MXene QD fluorescent probes for selective Fe^3+^ detection.^[^ ^213^ ^]^ The internal mechanism is based on the oxidation‐reduction reaction between the MXene and Fe^3+^, and the internal filtering effect. Similarly, Cd(II) ions in water can be pre‐concentrated and self‐reduced to Cd metal on Ti_3_C_2_T*_x_* MXene. Then the re‐oxidation signal can be measured in differential pulse voltammetry (DPV) technology,^[^ ^90^ ^]^ and the limit of detection was low as 0.94 × 10^−9^ m.

Applications in therapeutic biomedicine are promising and may be achieved with the rational designs of MXene‐based composites.^[^ ^214^, ^215^, ^216^ ^]^ Dai et al. prepared MnO*_x_*/Ti_3_C_2_ hybrids by the in situ reduction of MnO_4_^−^ ions on a Ti_3_C_2_ surface.^[^ ^214^ ^]^ As shown in Figure 13c, the Ti_3_C_2_ acts as the photothermal‐transducing agent for tumor ablation due to the high photothermal‐conversion efficiency of MXene. MnO_x_ acts as magnetic resonance (MR) and photoacoustic (PA) imaging contrast agents to determine tumor sites and monitor therapeutic process. In vitro and in vivo PA‐imaging of MnO*_x_*/Ti_3_C_2_ are shown in Figure 13d. Exposure to an 808 nm laser with sufficient power density, was highly efficient in causing the tumor ablation and growth suppression.

## 4 Summary and Prospects

This review has summarized progress on the roles of metal ions in MXene synthesis, processing and applications. Based on the way they change the MXene structure, three classifications are defined: intercalation, crosslinking, and etching. The understanding of the fundamental mechanisms has also been discussed. In the case of intercalation and crosslinking, electrostatic attraction between the metal cations and negatively charged surface groups is the driving force, and this ion exchange behavior enables the insertion of other metal ions. The hydration of metal cations plays a crucial role in improving the exfoliation kinetics and changing the interlayer spacing. Hydrated metal ions intercalate the MXene layers, rather than bare metal ions, and a large hydrated radius tends to produce a large c‐LP. Metal ions with high charge tend to form strong chemical or ionic bonds with the surface groups on adjacent MXene sheets. This crosslinking by metal ions induces MXene gelation to form 3D structures, with a sufficiently high MXene concentration. In the case of etching, the charge transfer seems to be the principal reason for the formation of in‐plane defects (Ti vacancies and in‐plane pores), due to the redox activities of the transition metal in MXenes. In the molten salt route to synthesize MXene, Ti atoms in MAX may also be etched, similar to the Al atoms, due to the higher redox potential (M*^x^* ^+^/M) of MCl*_x_* salts. The energy level differences between them determine the degree of charge transfer. The quality of MXene highly depends on the choice of molten salts. In addition, the Ti vacancies can anchor reduced M atoms to form single‐atom metals on MXene lattices.

The applications, by using the interactions and some targeted designs, have also been demonstrated, mainly in supercapacitors, metal‐ion batteries, catalysis, and water treatment. Energy storage in MXenes is closely related to the valence of transition metal atoms. In supercapacitors with neutral electrolytes, the hydration of metal cations is thought to hinder charge transfer between intercalated cation and MXenes. As a result, only capacitive behavior is seen. In metal‐ion batteries, de‐solvation and pillaring are thought to benefit ion intercalation and charge transfer. To improve the electrochemical performance, interlayer structure control, 3D structures, and MXene‐based metal hybrids produced with the assistance of metal ions are used. MXene‐supported single metal atom catalysts and a range of MXene metal hybrids can be prepared by the in‐situ reduction or derivation of metal ions on MXenes. MXenes demonstrate excellent removal efficiency of heavy metal ions and ion sieving activities through their strong interactions with metal ions, which plays a vital role in water/wastewater treatment.

Research on interactions between metal ions and MXenes is of great importance in MXene synthesis, processing, and applications. However, there are still many issues and scientific questions to be resolved. First, research to date has mostly been focused on the Ti_3_C_2_ synthesized by the HF or LiF/HCl methods, and interactions with the same metal ions are predicted to vary with the compositions and surface terminations of MXenes. In this regard, more relevant theoretical and experimental research is urgently needed. Second, for alkali metal ions, hydration (solvation) is crucial to open the MXene interlayers but seems to hinder charge transfer, which may reduce the capacitance/capacity in energy storage. A deeper understanding of their relationships is required to explore the potential of MXenes as electrode materials. Third, the MXene crosslinking by metal ions is innovative and effective for producing 3D structure. However, relevant research is limited, and more effort is needed to regulate structural parameters, including pore size and layer thickness. Fourth, in MXene evolution from MAX phases, the kinetics of etching reactions is a crucial issue to obtain MXenes of high quality and few defects. For etching Ti_3_AlC_2_ in HF/LiCl, this is addressed by the strong bonding of Al‐F and hydrated Li^+^ intercalation. However, the strategy of Li^+^ intercalation seems to play a limited role in other MXene syntheses, such as MoC_2_, Cr_2_C, or some o‐MXenes (e.g., Ti_2‐_ *_y_* Nb*_y_* CT*_x_*, V_2‐_ *_y_* Nb_y_CT*_x_*), which cannot be obtained in HF/LiCl. As such, there may be other important factors, and new synthesis routes for MXenes need to be investigated. Considering our current knowledge of the importance of etching and intercalation in the synthesis process, may be other ways of regulating the structure.

## Conflict of Interest

The authors declare no conflict of interest.
